# Empirical Evaluation of an Elitist Replacement Strategy for Differential Evolution with Micro-Populations

**DOI:** 10.3390/biomimetics10100685

**Published:** 2025-10-12

**Authors:** Irving Luna-Ortiz, Alejandro Rodríguez-Molina, Miguel Gabriel Villarreal-Cervantes, Mario Aldape-Pérez, Alam Gabriel Rojas-López, Jesús Aldo Paredes-Ballesteros

**Affiliations:** 1 Centro de Innovación y Desarrollo Tecnológico en Cómputo, Red de Expertos en Robótica y Mecatrónica, Instituto Politécnico Nacional, Mexico City 07700, Mexico; ilunao1900@alumno.ipn.mx (I.L.-O.); maldape@ipn.mx (M.A.-P.); arojasl2101@alumno.ipn.mx (A.G.R.-L.); jparedesb1900@alumno.ipn.mx (J.A.P.-B.); 2 Colegio de Ciencia y Tecnología, Universidad Autónoma de la Ciudad de México, Mexico City 06720, Mexico

**Keywords:** differential evolution, micro evolutionary algorithm, optimization, elitist mechanism, controller tuning

## Abstract

This paper introduces a variant of differential evolution with micro-populations, called μ-DE-ERM, which incorporates a periodic elitist replacement mechanism with the aim of preserving diversity without the need to measure it explicitly. The proposed algorithm is designed for scenarios with reduced evaluation budgets, where efficiency and convergence stability are critical. Its performance is evaluated on CEC 2005 and CEC 2017 benchmark suites, covering unimodal, multimodal, hybrid, and composition functions, as well as on two real-world engineering problems: the identification of dynamic parameters and the tuning of a PID controller for a one-degree-of-freedom robotic manipulator. The comparative analysis shows that μ-DE-ERM achieves competitive or superior results against its predecessors DE and μ-DE, and remains effective when contrasted with advanced algorithms such as L-SHADE and RuGA. Furthermore, additional comparisons with algorithms with competitive replacement mechanisms, μ-DE-Cauchy and μ-DE-Shrink, confirm the robustness of the proposal in real applications, particularly under strict computational constraints. These findings support μ-DE-ERM as a practical and efficient alternative for optimization problems in resource-limited environments, delivering reliable solutions at low computational cost.

## 1. Introduction

Nowadays, a wide variety of issues of interest in science and technology can be modeled, through mathematical language, as optimization problems. In these problems, the aim is to improve or optimize the performance of an associated system by adjusting the values of the variables that determine how it operates, also known as design variables. These problems require the minimization of one or more objective functions that quantify relevant measures of the system, such as costs or benefits, and are subject to a set of constraints that represent its physical, technical, or operational limitations. The solution to a problem of this type consists of finding configurations of the values of the design variables that satisfy the imposed constraints while minimizing the values of the objective functions. Many of these problems can be easily found in various fields of knowledge, such as engineering [1], architecture [2], health sciences [3], administrative sciences [4], and physics [5], to name a few, and have characteristics that make them difficult to solve analytically [6]. Among the main challenges are highly nonlinear, discontinuous, non-differentiable, or noise-affected objective functions and constraints [7]; a large number of design variables [8]; or the presence of mixed variables, both continuous and discrete [8]. In addition, certain conditions inherent to the problem, such as dynamism in the environment, multi-modality of the search space, or the existence of trade-offs between objectives [9,10], further increase the complexity.

Fortunately, metaheuristics based on evolutionary computation and swarm intelligence, in special those that are supported by rigorous mathematical analysis and widely recognized within the scientific community, offer an effective alternative for tackling difficult optimization problems, in the sense of the challenges mentioned above [11]. These techniques can find good approximate solutions without relying on strict mathematical properties of the problem and, in general, do so at a reasonable computational cost [12].

Today, there is a wide variety of metaheuristics aimed at solving problems that present particular challenges. Each metaheuristic usually draws inspiration for its operation from phenomena observed in nature, such as the natural evolution of species, the collaborative behavior of swarms in the search for resources for their subsistence and in the evasion of threats, the interactions between groups of individuals to achieve a particular goal, and other physical, biological, and social processes [13].

Among the huge variety of metaheuristics that exist today, differential evolution (DE) [14] stands out as one of the most effective alternatives for solving global optimization problems. In DE, a population is started with a set of individuals, which represent candidate solutions to an optimization problem. This initial population is randomly generated within the search space, defined by the lower and upper limits of each design variable. Over a pre-established number of generations, the population evolves through specific mutation and recombination operators specific to the DE algorithm, which generate new individuals called offspring. In each generation, these offspring compete with the original individuals based on their fitness, i.e., their ability to minimize the objective functions while satisfying the constraints of the problem. Those with the best performance are selected to form the next generation. At the end of the evolutionary process, the population contains the best individuals found, which constitute good approximations to the optimal solution to the problem.

The previous operation of DE is known for being simple and efficient, as well as competitive in solving challenging real-world problems, and is often used as a benchmark in solving various optimization problems [15]. That is why recent uses of DE can be found in robot tracking control [16], in the adjustment of neural networks for tumor detection [17], in camera calibration [18], in feature selection for respiratory disease analysis [19], in energy efficiency applications [20], among many others. In each of these applications, DE undergoes modifications, new mechanisms, or improvements in its operators to effectively address the particular characteristics of each optimization problem.

In some optimization problems derived from real-world applications, limitations exist in computing resources or time available for evaluating the problem or searching for a solution using algorithms like DE. Likewise, many of these applications require that solutions be found within narrow time windows. An example of such applications can be found in the adaptive tuning of controllers based on online optimization using metaheuristics [21,22], where solutions to the tuning problem must be found within very short time intervals. These limitations also arise in optimization problems associated with route planning in mobile robots [23], where the onboard computing equipment, usually embedded, tends to have reduced processing capacity, which must be distributed among multiple tasks to maintain the system’s autonomy. This constraint can significantly limit the number of evaluations of the problem, even if it is relatively simple, during its solution with metaheuristics. Similarly, in applications where the optimization problem is too extensive or complex, very time-consuming to evaluate, highly resource-intensive, or intermittently available, it is necessary to evaluate it as few times as possible. An example of this can be found in the optimization of hyperparameters of complex machine learning models [24].

One strategy that can be adopted in a metaheuristic to address the above limitations is to adjust the algorithm parameters to reduce the number of function evaluations required to find a suitable solution. In this regard, one can choose to reduce the population size in population-based algorithms such as DE, or the number of iterations/generations it uses. However, the first alternative is more common, as it allows the evolutionary dynamics of the algorithm to be preserved, i.e., the process of generating new promising solutions, without prematurely truncating the search process. Metaheuristics that use a small number of individuals are commonly known as micro evolutionary algorithms (μ-EAs). Although there is no clear consensus, a micro algorithm is usually considered to be one that uses a population of up to ten individuals [25,26,27].

At this point, it is important to mention that reducing the size of the population decreases the diversity among solutions, which can hinder exploration and favor premature convergence of the algorithm toward local solutions. Therefore, μ-EAs adopt mechanisms dedicated to increasing diversity, which are commonly used when it is detected that diversity has declined. The metrics used to estimate population diversity in this sense are generally based on measures of distance between solutions in the population [28]. On the other hand, among the mechanisms for preserving diversity is the elitist reinitialization of the population, which consists of replacing a portion of the individuals, usually at random, while preserving the most promising solutions [29]. In this way, diversity is reintroduced without completely restarting the evolutionary process, allowing the progress of the search achieved so far to be maintained. Another way to maintain diversity is by including random perturbations in the current solutions [30]. Depending on the magnitude of the disturbance, exploration or exploitation can be favored in the algorithm. For their part, dispersion mechanisms help distribute the population’s solutions to promising regions of the search space, reducing the probability of premature convergence [31]. In a different approach, previously found solutions are stored in a file for reuse in the current population with the intention of increasing its diversity [32], either to replace solutions or to redirect them to particular regions of the search space, both known and unexplored. Another type of mechanism is used to continuously modify the hyperparameters of the algorithm during the evolutionary cycle, helping to increase its exploratory ability [33]. Alternatively, mechanisms can be used to initialize the algorithm’s population with the greatest possible diversity, with the intention of gradually decreasing it over generations [34].

Despite the variety of strategies proposed in the literature, most diversity-preservation mechanisms for μ-EAs either rely on explicit monitoring, require additional parameter tuning, or introduce significant computational overhead. This creates a gap when algorithms must operate under strict evaluation budgets or in real-time and embedded scenarios, where such complexity is impractical. The specific problem addressed in this work is how to maintain diversity and prevent premature convergence in μ-EAs without additional monitoring or costly mechanisms, while still preserving the progress of the evolutionary process.

In this paper, an elitist periodic replacement mechanism is proposed as a simple and effective strategy for preserving diversity in differential evolution algorithms with micro-populations. The mechanism consists of randomly replacing a portion of the population every certain number of generations, while retaining the best solutions found up to that point. Unlike other approaches that require monitoring diversity to activate recovery mechanisms, the proposed strategy acts regularly and without the need for additional measurements, which simplifies its implementation. In this way, diversity is reintroduced in a controlled manner, without completely restarting the evolutionary process, which helps mitigate premature convergence and maintain the progress of the search. The effectiveness of the mechanism is evaluated empirically through tests on standard benchmark functions, and its effectiveness is demonstrated in a real application involving the identification of dynamic systems and the tuning of controllers. As the use of micro-populations introduces an intentional constraint on computational effort that mirrors the limitations of real-time or embedded systems, micro-evolutionary algorithms are designed specifically for environments where fast convergence and minimal resource usage are essential. Thus, their utility extends not only to efficiency but also to enabling optimization in contexts where standard population sizes and long trial runs are simply not possible. This makes the present study necessary, as they offer practical advantages, particularly in dynamic or resource-limited applications such as robotics or online controller tuning.

The contributions of this work are the proposal of a variant of differential evolution with micro-populations, called μ-DE-ERM, which integrates a periodic elitist restart mechanism to preserve diversity without the need to monitor it explicitly; the development of an exhaustive comparative analysis not only against standard DE and μ-DE, but also against more advanced algorithms such as L-SHADE and RuGA, as well as alternative micro-population strategies (μ-DE-Cauchy and μ-DE-Shrink), over benchmark functions with different levels of difficulty; the practical application of the proposal in two real engineering problems, namely the identification of dynamic parameters and the tuning of a PID controller for a one-degree-of-freedom robotic manipulator; and the detailed discussion of the trade-off between exploitation, favored by elitism, and exploration, enhanced by partial restart, showing that the methodology achieves an effective balance and offers a robust and competitive alternative to solve optimization problems in scenarios with limited computational resources.

The rest of the paper is organized as follows. Section 2 describes in depth the differential evolution algorithm and its best-known variants. Section 3 presents the elitist periodic replacement mechanism and how it is included within differential evolution. The comparative results showing the effectiveness of the variant derived from this algorithm are presented in Section 4, and the conclusions are included in Section 5.

## 2. Differential Evolution

The differential evolution algorithm [14] was originally designed to solve unconstrained global optimization problems in continuous search spaces in the form of (1) and (2). In (1), $p\in R^{n}$ is the vector of continuous design variables and J(p) is the scalar objective function. On the other hand, the search space is bounded in (2) by the lower limits $p_{l}$ and upper limits $p_{u}$ of the design variables in *p*.

(1) $$\min_{p\in R^{n}}\;J\left(p\right)$$

subject to:

(2) $$p_{l}\leq p\leq p_{u}$$

Using the optimization problem in (1) and (2), DE can approximate good solutions through the process described by Algorithm 1. In addition to the problem information, i.e., the objective function *J* and the search space limits $p_{l}$ and $p_{u}$, this algorithm requires several hyperparameters such as the maximum number of generations/iterations $G_{max}$ in which the evolutionary operators of mutation, crossover, and selection will be used on each individual in the fixed-size population NP. Additionally, the mutation operator requires the scaling factor *F*, while recombination uses the crossover probability CR. At the beginning of the algorithm, i.e., in the first generation G=1 (line 1), an initial population *P* with NP individuals is generated, commonly distributed randomly within the search space delimited by $p_{l}$ and $p_{u}$ (lines 1 and 2). These initial solutions evolve during the iterative cycle of $G_{max}$ generations (lines 3 to 8). In each generation *G* and for each of the individuals $p_{i}$ in the population *P* (line 4), a mutant $v_{i}$ is generated using different parents selected from *P* and the scaling factor *F* (line 5). Subsequently, the mutant $v_{i}$ recombines with the original individual $p_{i}$ to give rise to an offspring $u_{i}$ (line 6). Then, the offspring $u_{i}$ competes with the original individual $p_{i}$ to determine, based on fitness, which of the two will remain in *P* (line 7). It should be noted that fitness is determined based on the information in the optimization problem, i.e., the solution’s feasibility and the performance function’s improvement. At the end of the algorithm, the population *P* will contain the fittest individuals found during the evolutionary cycle. From among these, the best can be selected as the approximate solution to the problem $p_{best}$ (line 9).
**Algorithm 1:** Differential evolution (DE)
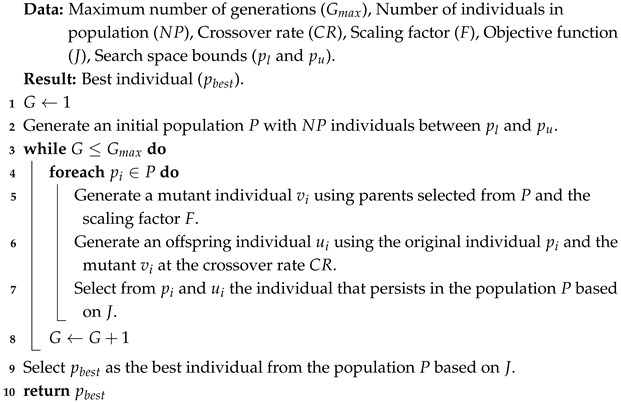


### 2.1. Variants of DE

Since the inception of DE, there have been different ways of performing the evolutionary operations of mutation and recombination. Each of these ways defines a different original variant of this algorithm and uses the nomenclature DE/x/y/z [14,35], where *x* and *y* indicate, respectively, the strategy and the number of differences between parent individuals used to generate the mutant individual. On the other hand, *z* refers to the operator used for the crossover of individuals. The variants of these evolutionary operators are described below.

#### 2.1.1. Mutation in DE

The original mutation operator of DE is presented in (3). For each individual $p_{i}$ in the population, the *i*-th mutant vector uses a base individual selected in some way from within the population. The base individual is perturbed by adding the *y* differences scaled by F∈[0,1] of different parent individuals randomly selected from the population $p_{r_{2}^{k}}-p_{r_{1}^{k}}$, with $i\neq r_{1}^{1}\neq r_{2}^{1}\neq \ldots \neq r_{1}^{y}\neq r_{2}^{y}$.

(3) $$v_{i}=\overset{\mathrm{Base}\;\mathrm{individual}}{\overbrace{p_{k}}}+F\underset{\mathrm{Parent}\;\mathrm{differences}}{\underbrace{\left(p_{r_{2}^{1}}-p_{r_{1}^{1}}+p_{r_{2}^{2}}-p_{r_{1}^{2}}+\ldots +p_{r_{2}^{y}}-p_{r_{1}^{y}}\right)}}$$

Regarding the selection of the base individual, Table 1 summarizes the original strategies used in DE. In this table, the mutation operator “rand” utilizes a base individual $p_{r_{3}}$ selected at random and distinct from the rest of the solutions used in the mutation. In the “best” operator, the base individual $p_{best}$ is the best solution in the current population. Finally, the “current-to-rand” and “current-to-best” operators use a base solution formed from the individual $p_{i}$ perturbed with the sum of the difference scaled by K∈[0,1], an additional scaling factor, between a random individual $p_{r_{3}}$ or the best in the population $p_{best}$, as appropriate, and the same individual $p_{i}$.

The above mutation variants differ in their exploratory capacity (i.e., to search for promising regions in the search space) and exploitative capacity (i.e., to search for promising solutions in particular regions of the same space). Strategies such as “rand” favor exploration, increasing the diversity of solutions in the population by performing stochastic combinations between individuals, but they can cause convergence to slow down. On the other hand, “best” alternatives, by directing the search toward the best known individual, have a greater capacity for exploitation, so they can accelerate convergence. However, this convergence could be premature toward a local minimum. This trade-off is well recognized in the literature [36,37]. Finally, the “current-to-rand” and “current-to-best” variants direct the search toward the current individuals, which allows them to be gradually adjusted and improved. However, there is a strong dependence on a high-quality initial population.

#### 2.1.2. Recombination in DE

Recombination in DE is carried out using two classic operators. These are applied independently to each design variable, denoted by *j*, which forms from both the individual $p_{i}$ and the mutant $v_{i}$.

The first of these operators is the binomial crossover, whose term *z* in the nomenclature corresponds to “bin”, and can be seen in (4). In this recombination strategy, a random number in [0,1] is generated for each design variable *j* with rand(0,1). If this number is less than the crossover rate CR, the corresponding design variable of the mutant $v_{i,j}$ is assigned to the same variable of the offspring $u_{i,j}$. Otherwise, it is assigned $p_{i,j}$. The same happens when $j=j_{rand}$, where $j_{rand}$ is a design variable selected at random before crossover, and guarantees that at least one of the variables of $v_{i}$ is inherited by $u_{i}$.

(4) $$u_{i,j}=\left\{\begin{array}{cc} v_{i,j} & \mathrm{if}\;rand\left(0,1\right)\leq CR\;\mathrm{or}\;j=j_{rand}\\ p_{i,j} & \mathrm{otherwise} \end{array}\right.$$

The second recombination operator is exponential crossover, indicated in DE nomenclature as “exp” and shown in (5). Unlike the “bin” operator, the crossover starts at a randomly selected index $j_{rand}$ and sequentially copies the design variables from $v_{i}$ to $u_{i}$ while rand(0,1)≤CR. Once this condition is no longer satisfied, the remaining variables of $u_{i}$ are inherited from the parent $p_{i}$. As in the “bin” strategy, the condition $j=j_{rand}$ is used to ensure that $u_{i}$ has at least one of the variables from $v_{i}$.

(5) $$u_{i,j}=\left\{\begin{array}{cc} v_{i,j} & \mathrm{from}\;j=j_{\mathrm{rand}}\;\mathrm{while}\;rand\left(0,1\right)\leq CR\\ p_{i,j} & \mathrm{otherwise} \end{array}\right.$$

**Figure 1:**
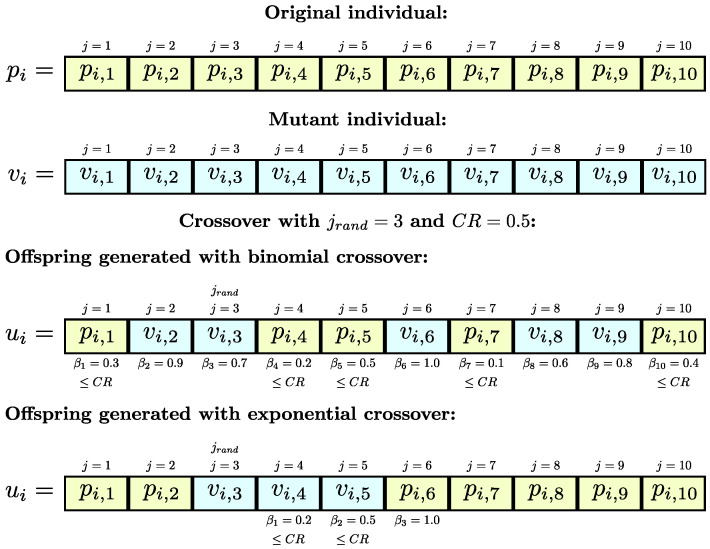
Example of how binomial and exponential crossover operators work in DE. The symbols $\beta_{k}$ correspond to values generated by rand(0,1), where the subscript *k* indicates the order in which they were obtained.

Figure 1 illustrates the operation of binomial and exponential crossover in DE for an example case with $j_{rand}=3$ and CR=0.5. In the binomial case, each component of the offspring is taken from either the mutant or the parent depending on the comparison between a random value $\beta_{k}$ and CR, while ensuring that the position $j_{rand}$ always comes from the mutant. In contrast, exponential crossover transfers consecutive variables from the mutant starting at $j_{rand}$ as long as $\beta_{k}\leq CR$, after which the remaining positions are inherited from the parent.

These recombination strategies determine how information from the mutant individual is inherited by the offspring. The binomial crossover “bin” helps distribute the mutant’s design variables throughout the offspring. This promotes greater diversity in the new solutions. In contrast, “exp” recombination promotes the inheritance of contiguous variables from the mutant. This decreases diversity in the population but favors controlled exploration and fine exploitation by preserving much of the original solutions when they are partially good, i.e., when the combination of some variables greatly impacts the minimization of the objective function.

## 3. Proposed Micro Differential Evolution with Elitist Restart Mechanism (μ-DE-ERM)

The DE variant proposed in this work, called micro differential evolution with elitist restart mechanism (μ-DE-ERM), uses a small population size to address optimization problems in which there are significant computational resource constraints or time limitations for executing the evolutionary process. This approach is particularly useful in applications that require dynamic or online optimization, which is carried out within embedded systems or computing equipment with limited resources, or in scenarios where function evaluation is costly and high-quality solutions must be obtained with a small number of evaluations.

This μ-DE-ERM is based on the variant presented in [38] and referred as μ-DE from now on, which is based on the DE/rand/1/bin strategy and uses a small population size (NP∈[3,6]). Nevertheless, the elitist restart mechanism is not restricted to this specific variant. It can be incorporated into other DE variants by adapting the mutation and recombination components accordingly. Presenting the DE structure with DE/rand/1/bin enables a clearer and more concrete understanding of how the proposed mechanism operates within the evolutionary cycle, offering several advantages, including improved exploration of the search space. To mitigate the loss of diversity associated with the small population size, a periodic and elitist restart mechanism is incorporated that preserves the best solutions found so far, preventing them from being lost during the restart process, while replacing the worst ones. This is activated each time a fixed number of generations elapses, known as generations for replacement (GR=100). At that point, a certain number of the worst individuals in the population (based on the value of the objective function), defined as replaced solutions (RP=2), are replaced by new solutions randomly generated in the search space. The purpose of this mechanism is to reintroduce variability into the population and reduce the risk of premature convergence.

The operation of μ-DE-ERM can be seen in Algorithm 2. There it can be observed that, like μ-DE, μ-DE-ERM uses DE/rand/1/bin (lines 1 to 3, and 6 to 12), since its mutation strategy selects base individuals randomly, which favors the exploration of the search space even with small populations. In addition, binomial recombination introduces changes distributed across multiple variables, promoting greater variability in the solutions generated. This combination allows for an adequate balance among simplicity, diversity, and computational efficiency, which are required in scenarios with limited resources. Similarly, μ-DE-ERM applies an elitist population reset mechanism every GR generations (lines 4 and 5). At this point, the main difference with respect to μ-DE lies in the use of the elitist restart mechanism (ERM) presented in Algorithm 3. The mechanism proposed in this work seeks to avoid the loss of valuable information accumulated during the evolutionary process by replacing the worst individuals with new solutions generated using information from other members of the population, rather than resorting to completely random solutions, as μ-DE does. To do this, the solutions in the population *P* are sorted in ascending order (from worst to best) with respect to their objective function value *J* (line 1). Then, for each solution $p_{i},\;i=1,\ldots ,RP$ that will be replaced, a replacement individual $p_{i}^{\prime }$ is generated using a mutation strategy similar to “best/1” (lines 2 and 3). In this sense, $p_{i}^{\prime }$ is created using the best individual in the current population $p_{best}$ as a basis, and this is perturbed with the difference of random parents chosen from among the worst solutions in *P*, with $w_{1},w_{2}\in \left[1,RP\right]$, $w_{1}=i$ and $w_{1}\neq w_{2}$ to ensure that the information from each of the worst solutions is involved in the creation of the individual that will replace $p_{i}$. At the end of the mechanism, the worst RP solutions in *P* are replaced by the previously generated individuals $p_{i}^{\prime }$ (line 4), and the updated population *P* is returned (line 5). The choice of a mutation strategy similar to “best/1” within the elitist restart mechanism allows the best current solution to be used as a basis, favoring the exploitation of knowledge acquired during the evolutionary process. By perturbing this solution using the difference between the worst individuals in the population selected at random, controlled diversity is introduced into the newly generated solutions. This allows the algorithm to obtain individuals that are not only promising but also sufficiently diverse, which helps to maintain the exploratory capacity without resorting to completely random solutions.
**Algorithm 2:** Proposed micro-differential evolution with elitist restart mechanism (μ-DE-ERM)
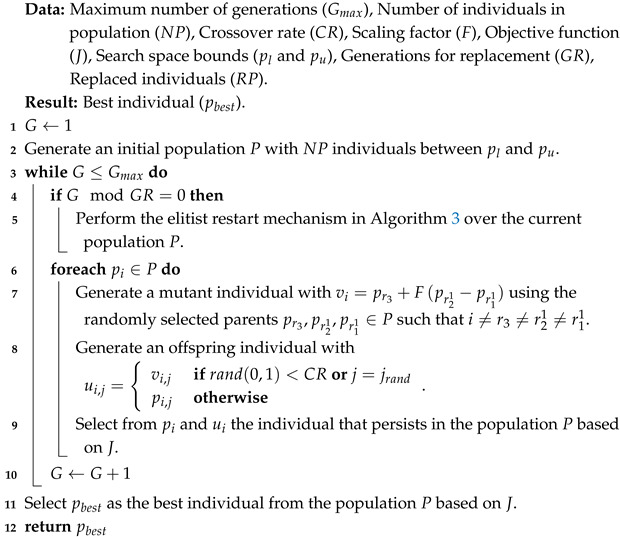


**Algorithm 3:** Elitist restart mechanism (ERM)

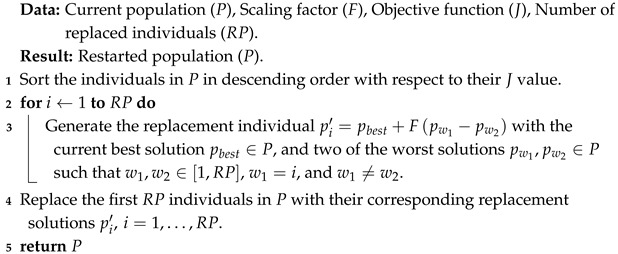



## 4. Comparative Study

This section presents an empirical study to evaluate the effectiveness of the proposed algorithm, μ-DE-ERM, in solving optimization problems with different levels of complexity. The analysis includes both benchmark functions with challenging characteristics and a real-world application case, corresponding to the identification and optimized tuning of a robotic system controller.

First, the results obtained with μ-DE-ERM over several benchmark problems are compared with those generated by its predecessor variants, i.e., μ-DE [38], the DE variant with micro-population and periodic random replacement of the worst solutions, and by the classic DE/rand/1/bin version (hereinafter referred to simply as DE), which uses a conventional population size. Next, more challenging benchmark problems are selected to compare μ-DE-ERM with advanced evolutionary algorithms that have shown high performance in the literature and represent contrasting approaches, on the one hand, parameter adaptation and progressive population reduction strategies, and on the other, micro-genetic schemes that emphasize efficiency with reduced populations. These are the variants of differential evolution with history-based parameter adaptation and linear population size reduction (L-SHADE) [39] and the real-coded micro-genetic algorithm (RuGA) [40], with linear crossover, non-uniform mutation, and tournament selection. Finally, μ-DE-ERM is compared with two other micro variants of DE, which, like this proposal, apply the reinitialization of the worst solutions in the population at fixed generation intervals, but differ in the competitive mechanisms used for such reinitialization. The first uses an adaptive strategy based on Cauchy deviates [41], which in this work is called μ-DE-Cauchy. The second generates new solutions randomly within a search space that progressively shrinks around a centroid defined by the current elite individual [42], and in this work, it is designated as μ-DE-Shrink.

### 4.1. Results of DE, μ-DE, and μ-DE-ERM over CEC 2005 Benchmark Optimization Problems

In these experiments, different budgets for function evaluations (FE) are considered. The hyperparameters used in each algorithm are detailed in Table 2, following the recommendations reported in [38]. It should be noted that the maximum number of generations is calculated as $G_{max}=FE/NP$, where NP is the population size. To obtain a statistically significant evaluation of the performance of each algorithm, 25 independent runs were performed per combination of problem and evaluation budget.

#### 4.1.1. Description of the CEC 2005 Benchmark Problems

The benchmark problems used to evaluate the performance of μ-DE-ERM and its predecessor variants, i.e., the base DE and the μ-DE, were taken from the definitions established in the special session on optimization with real parameters at the Congress on Evolutionary Computation 2005 (CEC 2005) [43]. This set of benchmark functions is widely used in the literature due to its scalability, diversity, and controlled level of difficulty [44,45,46], making it a suitable tool for evaluating the performance of algorithms with micro-populations, as it allows their effectiveness to be analyzed under limited computational conditions.

Eleven minimization functions with different challenging characteristics were used as the basis for the experimental evaluation of μ-DE-ERM, which are summarized in Table 3. Additionally, eleven shifted problems are generated by subtracting a constant vector $s\in R^{D}$ from the vector of design variables $p\in R^{D}$, and adjusting the vertical value of the objective function $f_{k}$ by adding a bias value $b_{k}$. This introduces new challenges to the problems by shifting the global optimum in both the search space and the minimum achievable value. In addition, other eleven problems are considered in which only a rotation is applied to the search space using the product R·(p−s), where $R\in R^{D\times D}$ is a linear transformation matrix. This is to evaluate the performance of the algorithm under non-separability conditions without displacement of the optimum. In summary, μ-DE-ERM is evaluated and compared against μ-DE and DE using a total of thirty-three optimization problems: the eleven base problems described in Table 3, the same eleven problems with global optimal displacement, and the same eleven problems with search space rotation. Full details of all problems are presented in Appendix A. The dimensionality of all problems is set to D=10. This choice allows it to evaluate the performance of the algorithm in a sufficiently challenging search space, but without excessively aggravating the loss of diversity characteristic of micro-populations. This prevents conclusions about the algorithm’s effectiveness from being distorted by excessively high dimensionalities, facilitating a more representative analysis of its actual behavior. Finally, each algorithm is assigned evaluation budgets of 1$\times 10^{3}$, 1$\times 10^{4}$, and 1$\times 10^{5}$ to solve each problem. This is done in order to analyze the quality of the solutions found under different computational constraints.

#### 4.1.2. Analysis of Descriptive Statistics on the CEC 2005 Benchmark Problems

Table 4, Table 5 and Table 6 present the descriptive statistics obtained when solving the thirty-three benchmark optimization problems using DE, μ-DE, and μ-DE-ERM, under different evaluation budgets of function evaluations (FE). In these tables, the first column corresponds to the FE values, the second to the algorithm used, the third to the statistical indicators considered (minimum, maximum, mean, and standard deviation), and the remaining columns show the corresponding values for each of the base, shifted, and rotated problems, respectively. The gray-shaded cells highlight the best value obtained among the algorithms for each statistic index considered, depending on the type of problem and FE. The findings from these three tables are as follows:

- Based on the results in Table 4, which considers problems that do not present displacement or rotation of the optimum, μ-DE-ERM showed solid performance with 1$\times 10^{3}$ evaluations. This behavior was especially evident in unimodal and non-separable functions such as $f_{2}$ and $f_{3}$, where it consistently outperformed DE and μ-DE. Even in separable functions such as $f_{1}$ and multimodal functions such as $f_{9}$, μ-DE-ERM achieved results close to the optimum. As the evaluation budget increased (up to 1 $\times \;10^{5}$ FE), DE dominated in mean and standard deviation, favored by its larger population, although μ-DE-ERM remained competitive in obtaining better minima. This suggests that its elitist mechanism allows it to preserve important evolutionary advances in early stages without requiring large computational resources.
- In the shifted problems analyzed in Table 5, μ-DE-ERM maintained its advantage for 1 $\times \;10^{3}$ FE, with the highest number of best results in all indicators, showing its ability to adapt efficiently to changes in the location of the optimum. However, with 1 $\times \;10^{4}$ and especially with 1 $\times \;10^{5}$ evaluations, DE showed a notable improvement, leading in mean and standard deviation, while μ-DE excelled in the minimums. Even so, μ-DE-ERM offered stable performance, indicating that its elitist restart mechanism allows it to maintain an acceptable level of performance even when the topological conditions of the problem are modified.
- Finally, in the rotated problems in Table 6, which eliminate separability and present greater difficulty due to the interaction between variables, μ-DE-ERM showed a good balance between exploitation and stability, standing out with 1 $\times \;10^{3}$ FE in the statistical indicators. Although μ-DE took the lead with 1 $\times \;10^{4}$ and 1 $\times \;10^{5}$ evaluations, μ-DE-ERM remained competitive, especially when finding values close to the optimum. This behavior indicates that, despite not leading in all indicators at high budgets, the μ-DE-ERM approach remains effective for exploring non-separable and multimodal spaces, such as those of $f_{6}$ and $f_{7}$, where it achieved outstanding performance with low and moderate budgets.

The best results presented in Table 4, Table 5 and Table 6 are summarized in Table 7 to facilitate analysis. In this table, the first three columns indicate the type of problem (base, shifted, or rotated), the FE values, and the corresponding algorithm. The next four columns report the number of times each algorithm obtained the best value in the statistical indicators considered (minimum, maximum, mean, and standard deviation) under each combination of problem type and FE. Finally, the last column shows the cumulative total of indicators in which the algorithm excelled. The findings extracted from this table are:

- In the base problems, μ-DE-ERM has a clear advantage with 1$\times 10^{3}$ evaluations, achieving the best values in 9 functions for the minimum, 5 for the maximum, 9 for the mean, and 6 for the standard deviation, accumulating a total of 29. This shows the effectiveness of its elitist mechanism under low-budget conditions, preserving promising solutions without losing diversity. With 1$\times 10^{4}$ FE, performance is balanced between μ-DE-ERM and DE (both with 16 counts), although with different profiles. In this case, DE dominates in mean and standard deviation, while μ-DE-ERM continues to lead in minimums. Finally, with 1$\times 10^{5}$ evaluations, DE consolidates its position as the best, achieving 28 counts and standing out in all indicators except minimums, where μ-DE and μ-DE-ERM tie with 8 each, although the rest of the counts favor DE.
- In displaced problems, μ-DE-ERM shows excellent performance with 1$\times 10^{3}$ FE, accumulating 9 best minimums, 7 maximums, 8 means, and 4 standard deviations, for a total of 28, far surpassing μ-DE (11) and DE (5). However, when the budget is increased to 1$\times 10^{4}$ evaluations, DE takes the lead with 29 counts, followed by μ-DE (16), while μ-DE-ERM drops to 10, indicating that its initial advantage is not maintained in this scenario. Finally, with 1$\times 10^{5}$ FE, DE and μ-DE show outstanding performance with 34 and 31 counts, respectively. DE dominates in maximum, mean, and std., while μ-DE stands out for its 11 best minimums, suggesting a more efficient ability to reach optimal solutions under extensive budgets. μ-DE-ERM, although stable, is behind with 20.
- In rotated problems, μ-DE-ERM leads with 1$\times 10^{3}$ evaluations, achieving 8 best minimums, 5 means, and 1 standard deviation, with a total of 17, slightly above μ-DE (15) and DE (12). By increasing FE to 1$\times 10^{4}$, μ-DE ranks as the best performing algorithm (20 counts), dominating the mean and standard deviation indicators, while μ-DE-ERM remains competitive with 18, thanks to its performance in minimums. Finally, with 1$\times 10^{5}$ evaluations, μ-DE maintains its advantage with 25 counts, showing a more balanced performance, while μ-DE-ERM (16) and DE (13) have a stable but less outstanding performance. This behavior indicates that μ-DE, despite its simplicity, adapts well to the complexity introduced by rotation when a larger computational budget is available.

#### 4.1.3. Analysis of Inferential Statistics on the CEC 2005 Benchmark Problems

Although the above results provide an overview of the performance of μ-DE-ERM, the stochastic nature of the algorithm requires a more rigorous analysis using inferential statistical techniques [47]. This need is reinforced by the Shapiro-Wilk normality test applied to the compared sample groups formed by problem type (base, shifted, and rotated), number of FE, and problem. Out of 99 comparison groups, only 20 showed normal distributions for the results of all three algorithms, which indicates that most cases deviate from normality (about 80%). For this reason, non-parametric statistical methods are required to draw strong conclusions. Therefore, the Kruskal-Wallis test was performed on the results obtained in the 25 independent and unpaired runs of the three compared algorithms, thus evaluating the existence of significant differences among them in each problem and budget of FE.

The test results are presented in Table 8, which shows the statistical value *H* of the compared groups (the larger the value, the greater the evidence of differences among the groups) and the *p*-value, which represents the probability of observing a difference equal to or greater than the one obtained, under the assumption that the null hypothesis $H_{0}$ is true. This hypothesis establishes that the compared groups have the same distribution. Therefore, to maintain high statistical significance, a decision threshold of α=0.05 is established, so that if *p*-value ≤α, $H_{0}$ is rejected. The shaded cells in the table indicate the cases where *p*-value ≤α.

Based on the results in Table 8, in most cases, *p*-values considerably lower than the significance threshold of α=0.05 were observed, indicating statistically significant differences among the results obtained by the evaluated algorithms. This is most clearly seen in the test scenarios that consider a smaller number of evaluations of the optimization problem (with 1$\times 10^{3}$ and 1$\times 10^{4}$ evaluations), where most functions have high *H* values and *p*-values close to zero, both in the base cases and in the shifted and rotated cases. When FE are increased to 1$\times 10^{5}$, some functions no longer show significant differences, e.g., $f_{8}$ and $f_{11}$, which could suggest that with a larger evaluation budget, the algorithms tend to perform similarly. The table also reveals that unimodal and separable functions such as $f_{1}$ tend to be solved more easily, while multimodal and non-separable functions such as $f_{6}$ to $f_{11}$ present greater challenges for the algorithms. These results indicate that the differences between algorithms are more pronounced when evaluation resources are limited, and that this behavior varies depending on the nature of the problem.

Since the Kruskal-Wallis test only indicates whether there are differences among all the algorithms, without specifying between which ones these differences occur, it is necessary to apply a post-hoc pairwise test to accurately identify those that are statistically significant. In this regard, the Mann-Whitney U pairwise test is used. The results of this post-hoc test, applied to the 25 independent samples obtained with each algorithm, are shown in Table 9. Each row of the table shows the comparison made by problem type, function, and evaluation budget. The reported results correspond to the *p*-values adjusted using the Holm-Bonferroni method. Similar to the Kruskal-Wallis test, the *p*-value is interpreted as the probability that the compared samples come from the same distribution. Statistical significance is also set at α=0.05. Each *p*-value in the table is preceded by the symbol “+” to indicate that the first test algorithm outperformed the second, “−” to indicate the opposite, and “≈” when there are no significant differences between the two. Cells that only include “−” indicate that the Mann-Whitney U test was not performed due to the results obtained previously with the Kruskal-Wallis test.

To make it easier to interpret the results of the Mann-Whitney U test, Table 10 summarizes the wins obtained by each algorithm. The outstanding results in this table are shaded. This table shows that the μ-DE-ERM variant obtained the highest number of wins in scenarios with low function evaluations (1$\times 10^{3}$), particularly in the base and shifted cases, highlighting its high performance under limited conditions. As the budget increases, the DE algorithm regains competitiveness, outperforming its variants in some scenarios, especially in the base and rotated problems. On the other hand, μ-DE maintains an intermediate and relatively stable performance in most cases. These results reinforce the trend previously observed in the Kruskal-Wallis global test, confirming that the differences among algorithms are consistent across multiple functions, and that the relative performance among them varies depending on the type of problem and the number of evaluations available.

Overall, the results on the CEC 2005 benchmark show that μ-DE-ERM is particularly effective with low budgets, where it achieves superior or comparable performance across all statistical indicators. Although its advantage decreases as FE increase, the algorithm continues to perform competitively and consistently compared to simpler or more populated variants, i.e., μ-DE and DE, respectively, supporting its applicability in optimization scenarios with limited resources.

### 4.2. Results of L-SHADE, RuGA, and μ-DE-ERM over CEC 2017 Benchmark Optimization Problems

As in previous experiments, different FE are used here. On the other hand, the hyperparameters of μ-DE-ERM match those in Table 2, with the exception of NP, which is set to 18 to handle the additional difficulties posed by the benchmark problems addressed in this section. As for L-SHADE and RuGA, they use the hyperparameters reported in [39] and [40], respectively. In each case, the maximum number of generations continues to be calculated as $G_{max}=FE/NP$, where NP is the population size of each algorithm. Likewise, 25 independent runs of each algorithm per combination of problem and evaluation budget are performed.

#### 4.2.1. Description of the CEC 2017 Benchmark Problems

In this stage, the benchmark problems used to evaluate μ-DE-ERM were some of those defined in the special session on single objective real-parameter optimization at CEC 2017 [48]. Compared to the CEC 2005 benchmark, this set incorporates more complex and diversified features, including hybrid and composite functions, which considerably increase the difficulty of the evaluation scenarios. For the comparisons with L-SHADE and RuGA, one representative function from each category in the benchmark suite (i.e., unimodal, simple multimodal, hybrid, and composition) was selected. The chosen functions are shown in Table 11 and provide a representative yet manageable subset, avoiding redundancy among functions with very similar properties and reducing the computational burden associated with evaluating the entire suite. As in the previous experiments, the algorithms use 1$\times 10^{3}$, 1$\times 10^{4}$, and 1$\times 10^{5}$ FE to solve each problem in order to analyze their performances under a variety of computational constraints.

#### 4.2.2. Analysis of Descriptive Statistics on the CEC 2017 Benchmark Problems

Table 12 presents the descriptive statistics obtained when solving the four benchmark optimization problems with L-SHADE, RuGA, and μ-DE-ERM, through 25 independent runs and considering different FE. The columns of this table include the FE value, the utilized algorithm, and the descriptive statistical indicators (minimum, maximum, mean, and standard deviation) for each problem. The best indicator values are gray-shaded for each test scenario given by FE. Based on this table, the following is observed:

- With a low budget of 1$\times 10^{3}$ evaluations, RuGA achieves the best minimums in the unimodal, multimodal, and hybrid functions ($f_{12}$ to $f_{14}$). At the same time, μ-DE-ERM stands out as the only one capable of consistently solving the composite function ($f_{15}$), always reaching the same value without variation, while L-SHADE remains clearly behind with much higher errors.
- By increasing to an intermediate budget of 1$\times 10^{4}$ evaluations, μ-DE-ERM consolidates its superiority by achieving values close to zero in $f_{12}$ to $f_{14}$ and maintaining stable accuracy in $f_{15}$, with almost zero deviations, while L-SHADE and RuGA show some improvement but continue to obtain results that are several orders of magnitude worse.
- With a high budget of 1$\times 10^{5}$ evaluations, μ-DE-ERM dominates in all functions, reaching practically zero values in $f_{12}$ to $f_{14}$ and the greatest stability in $f_{15}$, where L-SHADE and RuGA fail to compete and present large errors. On the other hand, although L-SHADE improves in simple and hybrid functions, it fails in the composite function, and RuGA loses competitiveness and consistency, suggesting that the proposal is more robust and stable as the budget increases.

To facilitate a clearer analysis, Table 13 summarizes the best results from Table 12. This table reports the number of times each algorithm was better in each statistical indicator for each FE scenario. The findings based on the table are listed next:

- At the lowest budget (1$\times 10^{3}$), RuGA initially dominates, although μ-DE-ERM already matches or exceeds its performance in several indicators.
- As the budget increases to 1$\times 10^{4}$, μ-DE-ERM clearly outperforms both competitors by achieving the majority of best values across all metrics, accumulating more than four times as many wins as L-SHADE or RuGA.
- Finally, at the highest budget (1$\times 10^{5}$), μ-DE-ERM and L-SHADE share the leading results, while RuGA loses competitiveness entirely. Overall, these results confirm that μ-DE-ERM is consistently competitive at low budgets and becomes the most reliable and effective approach as the number of evaluations increases.

#### 4.2.3. Analysis of Inferential Statistics on the CEC 2017 Benchmark Problems

Like the other algorithms evaluated previously in this study, L-SHADE and RuGA are stochastic in nature. Therefore, the Shapiro-Wilk test was applied to the sample groups formed by FE and problem to assess normality. The results showed that 100% of the distributions in the compared groups (12 in total) were non-normal, which makes it necessary to use a non-parametric statistical method. Therefore, the Kruskal-Wallis test is also performed here on the results of the 25 independent and unpaired runs of μ-DE-ERM, L-SHADE, and RuGA on the CEC 2017 problems. This is done to observe whether there are significant differences among these three algorithms when different FE are considered. The results of this test are in Table 14, which includes the statistical value *H* of the compared groups and the *p*-value. The decision threshold of α=0.05 is also considered, and the gray-shaded cells indicate that the *p*-value ≤α, i.e., that the groups have different distributions. According to the results in Table 14, all the groups of samples, given by the problem and the FE, have significant differences, with *p*-values quite far from α. Consequently, a post-hoc test is required to identify the source of these differences.

The Mann-Whitney U pairwise test is also used here to determine the dominant alternatives of each pair of algorithms for each problem and evaluation budget. The results are included in Table 15. This table presents the *p*-values of each test, adjusted by the Holm-Bonferroni method. The symbol “+” is shown before the value to indicate that the first test algorithm outperformed the second, “−” to indicate the opposite, and “≈” when there are no significant differences (according to a threshold α=0.05).

The results from this post-hoc test in Table 15 are summarized in Table 16 for ease of interpretation. This last table shows the wins achieved by each algorithm for each problem and FE. The results in this table reveal that μ-DE-ERM consistently achieves the highest number of victories across the CEC 2017 problems, particularly at medium and high evaluation budgets. At 1$\times 10^{3}$ evaluations, RuGA initially obtains more wins, although μ-DE-ERM still performs competitively. When the budget increases to 1$\times 10^{4}$, μ-DE-ERM clearly dominates with six victories, doubling the counts of L-SHADE and RuGA. At 1$\times 10^{5}$ evaluations, both μ-DE-ERM and L-SHADE are competitive, but the proposed algorithm retains the highest overall win count, confirming its robustness and superiority in more demanding scenarios.

In summary, the experiments on the CEC 2017 benchmark show that although RuGA is competitive at very low budgets and L-SHADE improves at higher ones, μ-DE-ERM consistently delivers the most stable and accurate results. The statistical analyses confirm that these advantages are significant, establishing μ-DE-ERM as the most reliable alternative under different computational budgets.

### 4.3. Results of μ-DE-Cauchy, μ-DE-Shrink, and μ-DE-ERM over a Real-World Problem

#### 4.3.1. Problem Description

The problems addressed in this section to evaluate the performance of the proposed DE variant in real-world scenarios correspond to two fundamental applications in dynamic systems: (1) system identification and (2) controller tuning.

Identification refers to the process by which a model can effectively generalize the behavior of a dynamic system is obtained, based on information from its inputs and outputs previously acquired by exciting as many of its dynamics as possible [49]. The adopted model has a mathematical or computational structure whose parameters are adjusted with the aim of minimizing differences with respect to the real system, i.e., the goal is that, when receiving the same inputs, both generate highly similar outputs. On the other hand, controller tuning consists of determining the parameters of the control system that regulate the behavior of the dynamic system in order to perform a specific task with high performance [50]. This tuning is usually done by simulating a model with which different combinations of parameters are tested, and the best ones are then implemented in the real system.

It should be noted that, currently, controller identification and tuning are often performed in constrained computing environments, either due to their integration into adaptive online control schemes [21,22] or their implementation in embedded or low-budget platforms [51]. Furthermore, by their very nature, system identification and controller tuning can be formulated as global optimization problems in the form of (1) and (2). Therefore, in this work, the problems of identification and control of a single-degree-of-freedom manipulator robot, focused on the task of position regulation, are addressed from the perspective of micro-population algorithms. The complete details of this system are shown in Appendix B, where the angular position and velocity of the robot are described by $\theta ,\;\dot{\theta }$, respectively. On the other hand, the aforementioned problems are presented below.

**Figure 2:**
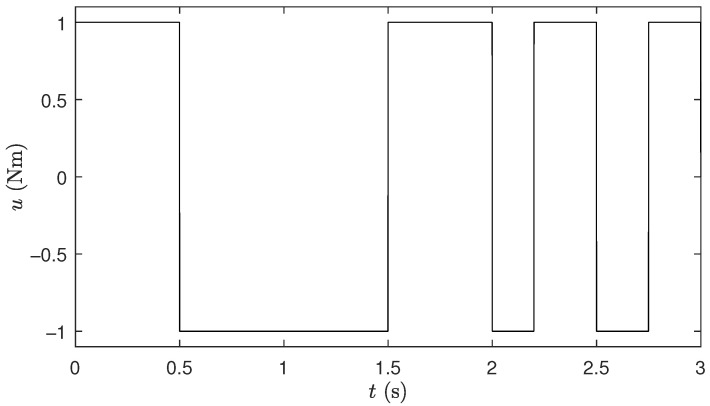
Predefined control signal $\bar{u}\left(t\right)$ utilized for system identification.

The problem of identifying the robotic manipulator presented in (6) and (7) consists of finding the parameters $p={\left[m,L_{cm},I_{z}\right]}^{T}$ of its dynamic model such that, when using the predefined control input $\bar{u}\left(t\right)$, the differences $e_{si}=\bar{y}\left(\bar{u},t+dt\right)-y\left(p,\bar{u},t+dt\right)$ between the outputs $\bar{y}\left(\bar{u},t+dt\right)={\left[\bar{\theta }\left(\bar{u},t+dt\right),\dot{\bar{\theta }}\left(\bar{u},t+dt\right)\right]}^{T}$, where $\bar{\theta }$ is the angular measured position, obtained from the real system (previously acquired) and the outputs $y\left(p,\bar{u},t+dt\right)={\left[\theta \left(p,\bar{u},t+dt\right),\dot{\theta }\left(p,\bar{u},t+dt\right)\right]}^{T}$ calculated with the model, are minimized. The above during an identification time window $t\in [0,t_{si}]$, with $t_{si}=3\;\left(s\right)$ and considering a sampling interval of dt=1(ms). The predefined control signal $\bar{u}\left(t\right)$ applied in the aforementioned time window is shown in Figure 2. The Integral Square Error (ISE) over $e_{si}$ is used as the objective function $J_{si}$ in (6) to measure these differences.

(6) $$\min_{p=\left[m,L_{cm},I_{z}\right]\in R^{3}}\;J_{si}\left(p\right)=\int_{t=0}^{t_{si}}e_{si}^{T}\;e_{si}\;dt$$

subject to:

(7) $$p_{l}\leq p\leq p_{u}$$

For its part, the controller tuning problem in (8) and (9) seeks the gains of the Proportional Integral Derivative (PID) controller, $g={\left[k_{p},k_{i},k_{d}\right]}^{T}$, which are used to calculate the control signal $\bar{u}\left(g,t\right)$ that governs the dynamics of the robotic manipulator model (known or identified) to minimize the error $e_{ct}=y_{d}-y\left(p,\bar{u},t+dt\right)$ in the position control task, with $y_{d}={\left[3\pi /4\;\left(\mathrm{rad}\right),0\;\left(\mathrm{rad}/s\right)\right]}^{T}$ as the desired output. As in the identification problem, ISE is used as the objective function $J_{ct}$ of (8) to measure the error in performing the predefined task during a simulation time window $t\in [0,t_{ct}]$, with $t_{ct}=5\;\left(s\right)$ and sampling interval dt=1(ms). The parameters of the model used for tuning are described in Appendix B.

(8) $$\min_{g=\left[k_{p},k_{i},k_{d}\right]\in R^{2}}\;J_{ct}\left(g\right)=\int_{t=0}^{t_{ct}}e_{ct}^{T}\;e_{ct}\;dt$$

subject to:

(9) $$g_{l}\leq g\leq g_{u}$$

In both optimization problems, the bounds of the box constraints (7) and (9) are set as in Table 17.

It is worth noting that, although in practice system identification and controller tuning are usually addressed sequentially, i.e., the system is first identified and then the model is used to tune the controller, in this work both problems are solved independently in order to ensure a fair comparison, giving all algorithms the same starting conditions. For the identification problem, the three algorithms use the same state measurements and open-loop input data from the system. For the tuning problem, it is assumed that the exact model is available to all algorithms. Therefore, this study does not consider a direct dependency between the two problems. Instead, they are treated as independent tasks whose solutions can be replicated or adapted individually.

#### 4.3.2. Analysis of Results for the Identification Problem

For the identification of the manipulator robot, each algorithm is assigned 3$\times 10^{3}$ FE, given that this is a highly complex problem in which it is necessary to adjust model parameters based on data measured from the actual system. The hyperparameters shared by μ-DE-Cauchy, μ-DE-Shrink, and μ-DE-ERM (i.e., the number of individuals NP, the scaling factor *F*, the crossover rate CR, the generations for replacement GR, and the replaced individuals RP) remain the same as the ones of μ-DE-ERM in Table 2. For μ-DE-Cauchy, a Cauchy scale of 0.15 is used to balance exploration and stability, while for μ-DE-Shrink, a shrink factor of 0.85 is chosen to progressively reduce the search space without losing diversity.

Table 18 shows the statistical summary of the results obtained after performing 25 independent runs of each algorithm on the system identification problem. This table shows the values of the design variables and the objective function for each run with each of the algorithms, sorted from lowest to highest with respect to the latter value. The bottom of the table shows the minimum, maximum, mean, and standard deviation values for each column. The best indicators with respect to the objective function are shaded in gray.

The descriptive results in Table 18 show that μ-DE-ERM obtained the best indicators for $J_{si}$, indicating a more accurate fit of the manipulator model. In addition, it exhibited lower variability among runs compared to μ-DE-Cauchy and μ-DE-Shrink, which suggests greater robustness and reliability. By contrast, μ-DE-Cauchy produced competitive solutions but with larger fluctuations due to the disruptive nature of the Cauchy perturbations, while μ-DE-Shrink showed more concentrated searches that reduced exploration, leading to higher residual errors in $J_{si}$. These results confirm the effectiveness of the proposed μ-DE-ERM for system identification problems when computational resources are limited.

**Figure 3:**
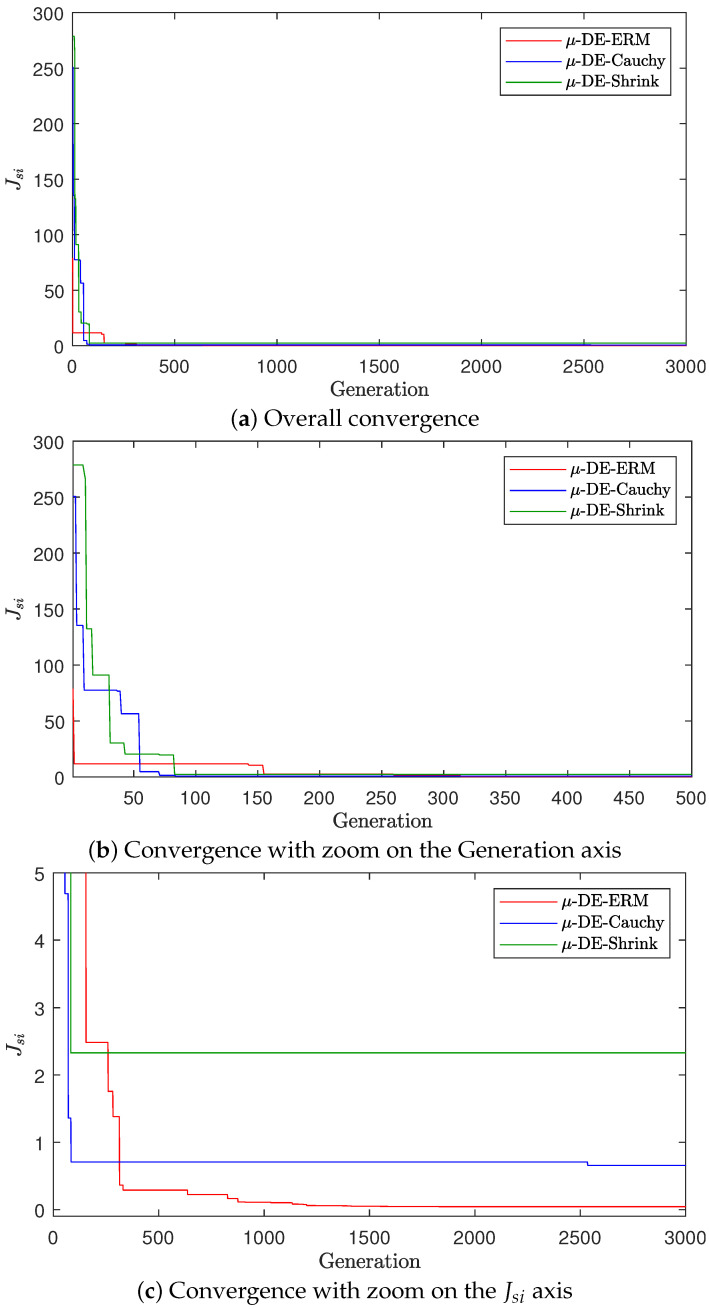
Convergence plots for the identification problem.

A convergence plot for the median run (with respect to $J_{si}$) is presented in Figure 3 to illustrate representative convergence behavior while mitigating the influence of outliers. The figure shows that μ-DE-ERM achieves faster and more stable reductions in the objective function compared to μ-DE-Cauchy and μ-DE-Shrink. Although all three variants exhibit similar initial trends, the median convergence curve of μ-DE-ERM consistently reaches lower final values, as highlighted in the zoomed-in views on the generation and $J_{si}$ axes. In contrast, μ-DE-Cauchy and μ-DE-Shrink exhibit earlier stagnation and remain at higher objective values, reflecting premature convergence and reduced accuracy in the identification task.

Additionally, it is of interest to analyze the computation time required by each algorithm to solve the identification problem. Table 19 reports the average total execution time and the mean time per generation. The experiments were carried out in MATLAB R2025a on a PC with an Intel(R) Xeon(R) CPU E5-2680 v4 at 2.40 GHz. As shown in the table, even in MATLAB, the algorithms require less than 6 (ms) per generation, which is sufficient for online identification since many applications only demand around ten generations [21,22]. This demonstrates that the proposed approach is already competitive and would be even faster in compiled implementations.

The above is confirmed by the Kruskal-Wallis test with α=0.05, which reveals significant differences among the three algorithms when solving the system identification problem, obtaining a *p*-value of 8.9490$\times 10^{-8}$ and an *H* value of 3.2458$\times 10^{1}$. It is worth noting that the Shapiro-Wilk test was also applied to the three sets of identification results obtained by the compared algorithms. According to the test, none of the samples exhibited normality. The results of the Mann-Whitney U post-hoc tests with the Holm-Bonferroni *p*-value adjustment and the same α=0.05 are shown in Table 20. The symbols preceding the *p*-value in the table indicate that there are no significant differences between the two algorithms (≈) or that the second algorithm outperformed the first (−). Based on these results, μ-DE-ERM is significantly better than the other two algorithms in solving the identification problem.

**Figure 4:**
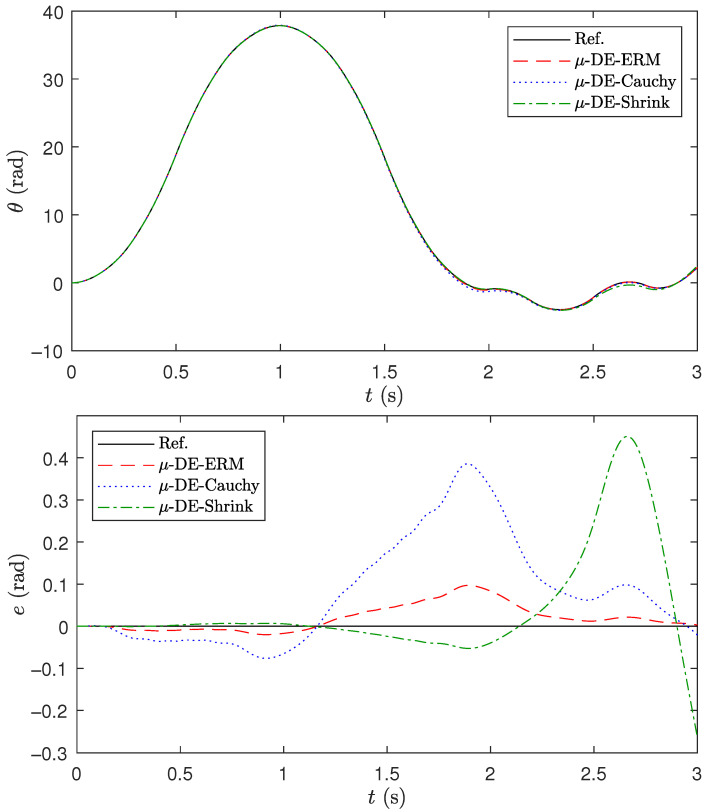
Manipulator system and model responses and errors when using a predefined $\bar{u}\left(t\right)$ and selected model parameters obtained with the different algorithms.

Figure 4 shows the response of the robotic manipulator and its error in contrast to that of the models adjusted with the different algorithms when they receive the same predefined control signal $\bar{u}\left(t\right)$ from Figure 2. The parameters of the models used in this sense correspond to those of the run with the median value of $J_{si}$ in Table 18. Here, it can be seen that the model adjusted by μ-DE-ERM generates an output that is closer to the actual one with less error.

#### 4.3.3. Analysis of Results for the Tuning Problem

In the case of PID controller tuning, 5$\times 10^{1}$ FE are used, as this is a simpler problem in which only three controller parameters are optimized on a known model. Due to this reduced number of function evaluations, an appropriate value of generations for replacement GR=10 is used for the three compared algorithms, i.e., μ-DE-Cauchy, μ-DE-Shrink, and μ-DE-ERM. The rest of their hyperparameters remain unchanged concerning those utilized for the identification problem.

Table 21 shows the descriptive results after 25 independent runs of each algorithm on the controller tuning problem. As in the case of the identification problem, the table presents the values of the design variables and the objective function for each run with the three algorithms, sorted in ascending order with respect to $J_{ct}$. The minimum, maximum, mean, and standard deviation values for each column are included at the end of the table. The best statistical indicators involving the objective function are shaded.

The descriptive results in Table 21 reveal that μ-DE-ERM consistently achieved the best indicators for $J_{ct}$, providing not only the lowest error but also the smallest variability among runs, which confirms its stability in finding high-quality PID gains. In comparison, μ-DE-Cauchy and μ-DE-Shrink reached feasible solutions but with higher dispersion, reflecting less robustness. Specifically, Cauchy produced competitive gain values but at the cost of greater fluctuations in performance, while Shrink yielded slightly better mean results than Cauchy yet with more variability than μ-DE-ERM. These outcomes validate the superiority of the proposed μ-DE-ERM in PID controller tuning tasks, especially under limited computational budgets.

**Figure 5:**
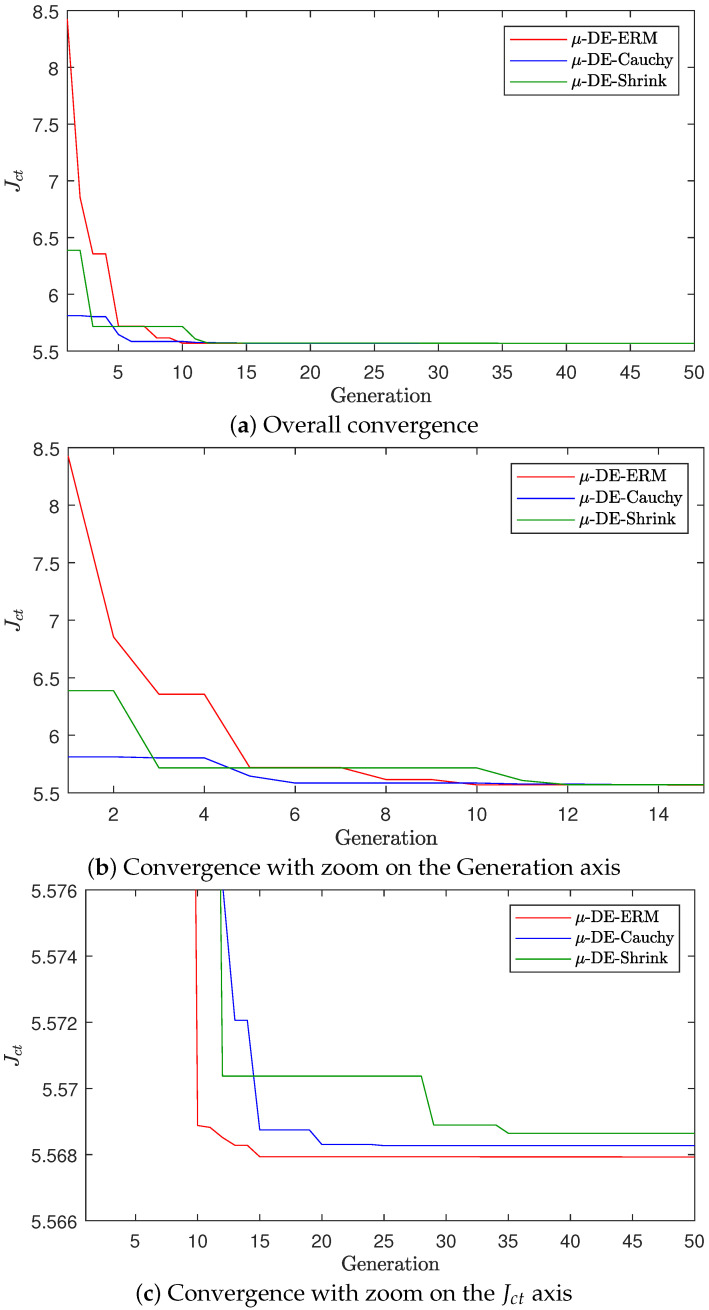
Convergence plots for the controller tuning problem.

A convergence plot for the median run (with respect to $J_{ct}$) is presented in Figure 5 to depict the representative behavior of the three algorithms while minimizing the influence of outliers. The results show that μ-DE-ERM achieves the most rapid and stable convergence toward lower objective values compared to μ-DE-Cauchy and μ-DE-Shrink. Although the three methods exhibit similar early convergence patterns, the median curve of μ-DE-ERM consistently reaches the lowest final $J_{ct}$ values, as emphasized in the zoomed-in views on the generation and $J_{ct}$ axes. Conversely, μ-DE-Cauchy and μ-DE-Shrink converge more slowly and stabilize at higher values, indicating reduced accuracy and less effective tuning performance.

The computation time is also analyzed here for the controller tuning problem. In this sense, Table 22 presents the average execution times for the controller tuning problem. The results show that all three algorithms require less than 5 (ms) per generation, which confirms their suitability for online tuning tasks. Such low computation times ensure that the optimization process can be integrated into real-time applications, where rapid response and efficient use of resources are essential.

Again, the Shapiro-Wilk test was applied to the three samples of controller tuning results obtained by the algorithms, and none of them exhibited normality. So, the Kruskal-Wallis test with α=0.05 is applied to the 25 results obtained by each algorithm, and significant differences are observed with a *p*-value of 1.2875$\times 10^{-9}$ and an *H* value of 4.0941$\times 10^{1}$. Mann-Whitney U post-hoc tests are also performed with the Holm-Bonferroni *p*-value adjustment and α=0.05. The results of these tests are presented in Table 23. In this case, the prefix “−” preceding the *p*-value indicates that the second algorithm outperformed the first, and “≈” suggests that there are no significant differences. The results of the post-hoc tests reveal that μ-DE-ERM significantly outperforms the other algorithms.

**Figure 6:**
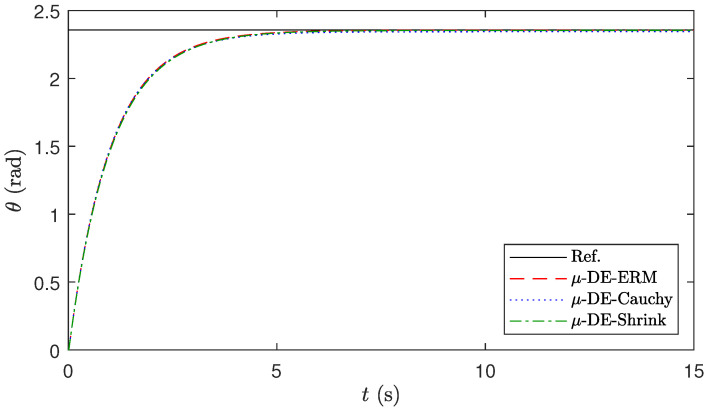

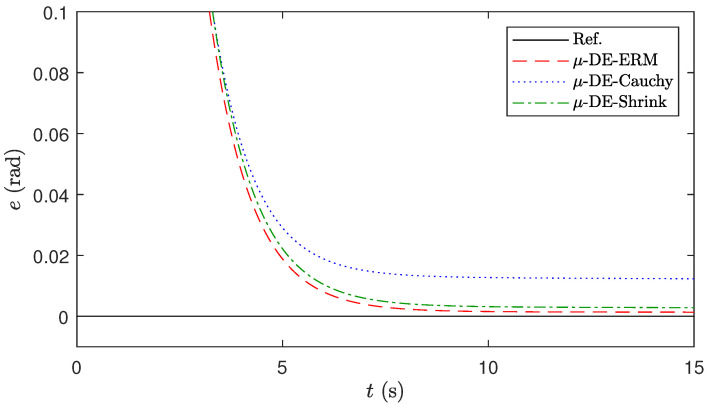
Manipulator response and error when using a selected PID controller parameters obtained with different algorithms.

Finally, Figure 6 shows the behavior and the error of the robotic manipulator in performing the position regulation task using different gains calculated with the three algorithms. These gains correspond to the runs with the median values of $J_{ct}$ in Table 21. In this figure, it can be seen that the controller tuned with μ-DE-ERM can take the position of the manipulator closer to the reference value in the simulated time window with less error.

## 5. Conclusions

The experimental results obtained in this work indicate that the μ-DE-ERM variant constitutes a significant improvement within evolutionary algorithms with micro-populations, thanks to the explicit incorporation of the periodic elitist replacement mechanism (ERM). This mechanism allows diversity to be maintained without the need to estimate or monitor it, which is particularly useful in environments with limited evaluation budgets, where traditional diversity control strategies can be costly or unfeasible.

In the benchmark experiments, μ-DE-ERM consistently outperformed DE and μ-DE, and demonstrated competitiveness against more advanced approaches such as L-SHADE and RuGA, particularly in complex multimodal, hybrid, and composition functions from the CEC 2017 suite. Moreover, the additional comparisons with μ-DE-Cauchy and μ-DE-Shrink highlighted that while these strategies provided useful alternatives for promoting exploration, μ-DE-ERM achieved a superior balance of exploitation and exploration, resulting in greater stability and robustness across diverse scenarios.

In applications involving the identification and tuning of controllers for a manipulator robot, μ-DE-ERM not only achieved better adjustment of dynamic and control parameters but also exhibited greater consistency in results. In particular, during identification, the mean error rate was significantly lower, and during PID controller tuning, the algorithm converged stably to optimal solutions in all runs. These results empirically validate that ERM acts as an evolutionary mechanism that increases the reliability of the algorithm under computationally constrained conditions.

Together, these findings support the use of the ERM mechanism as an effective and low-cost solution for preserving diversity in evolutionary micro-algorithms, allowing for an efficient balance between exploration and exploitation. Its simple implementation, together with the observed benefits, make μ-DE-ERM a promising tool for solving optimization problems in real time, online, or on embedded platforms where the evaluation budget is limited by hardware or execution speed constraints.

Future work will explore extending the proposal to adaptive controller tuning, where identification and control parameters must be optimized jointly within short time windows, as well as investigating hybridization with parameter adaptation schemes to further enhance robustness in dynamic environments. In addition, a detailed sensitivity analysis of the algorithm’s performance with respect to hyperparameter variations will be conducted to provide further insights and practical recommendations.

## Figures and Tables

**Table 1:** Mutation operators in DE considering *y* differences of parent individuals.

Mutation Strategy (x/y)	Operator
rand /y	$v_{i}=p_{r_{3}}+F\;\sum_{k=1}^{y}\left(p_{r_{2}^{k}}-p_{r_{1}^{k}}\right)$
best /y	$v_{i}=p_{best}+F\;\sum_{k=1}^{y}\left(p_{r_{2}^{k}}-p_{r_{1}^{k}}\right)$
current-to-rand /y	$v_{i}=p_{i}+K\;\left(p_{r_{3}}-p_{i}\right)+F\;\sum_{k=1}^{y}\left(p_{r_{2}^{k}}-p_{r_{1}^{k}}\right)$
current-to-best /y	$v_{i}=p_{i}+K\;\left(p_{best}-p_{i}\right)+F\;\sum_{k=1}^{y}\left(p_{r_{2}^{k}}-p_{r_{1}^{k}}\right)$

**Table 2:** Hyperparameters of μ-DE-ERM and its predecessor variants.

Hyper-Parameter/Algorithm	DE	μ-DE	μ-DE-ERM
Number of individuals in population (NP)	30	6	6
Scaling factor (*F*)	0.9	0.9	0.9
Crossover rate (CR)	0.1	0.1	0.1
Generations for replacement (GR)	−	100	100
Replaced individuals (RP)	−	2	2

**Table 3:** Base benchmark problems from CEC 2005.

Problem	Name	Features
$f_{1}$	Sphere Function	Unimodal, separable, scalable
$f_{2}$	Schwefel’s Function No. 1.2	Unimodal, non-separable, scalable
$f_{3}$	Elliptic Function	Unimodal, non-separable, scalable
$f_{4}$	Schwefel’s Function No. 1.2 with noise	Unimodal, non-separable, scalable, noise
$f_{5}$	Schwefel’s Function No. 2.6	Unimodal, non-separable, scalable
$f_{6}$	Rosenbrock’s Function	Multi-modal, non-separable, scalable
$f_{7}$	Griewank’s Function	Multi-modal, non-separable, scalable
$f_{8}$	Ackley’s Function	Multi-modal, non-separable, scalable
$f_{9}$	Rastrigin’s Function	Multi-modal, separable, scalable
$f_{10}$	Weierstrass Function	Multi-modal, non-separable, scalable
$f_{11}$	Schwefel’s Problem No. 2.13	Multi-modal, non-separable, scalable

**Table 4:** Results obtained with DE, μ-DE, and μ-DE-ERM over the base CEC 2005 benchmark problems after 25 independent runs, considering evaluation budgets of 1$\times 10^{3}$, 1$\times 10^{4}$, and 1$\times 10^{5}$.

FE	Algorithm	Index	Base $f_{1}$	Base $f_{2}$	Base $f_{3}$	Base $f_{4}$	Base $f_{5}$	Base $f_{6}$	Base $f_{7}$	Base $f_{8}$	Base $f_{9}$	Base $f_{10}$	Base $f_{11}$
1$\times 10^{3}$	DE	Min	1.0546$\times 10^{3}$	2.8034$\times 10^{3}$	6.6370$\times 10^{5}$	2.7731$\times 10^{3}$	9.8700$\times 10^{4}$	5.5282$\times 10^{6}$	9.2782	1.1302$\times 10^{1}$	8.5156$\times 10^{1}$	5.8456	1.6902
		Max	2.5543$\times 10^{3}$	6.8417$\times 10^{3}$	1.8393$\times 10^{7}$	8.7422$\times 10^{3}$	1.7100$\times 10^{5}$	1.3026$\times 10^{8}$	2.7038$\times 10^{1}$	1.5578$\times 10^{1}$	1.3272$\times 10^{2}$	8.9490	4.1295
		Mean	1.7063$\times 10^{3}$	4.8810$\times 10^{3}$	8.1794$\times 10^{6}$	6.2957$\times 10^{3}$	1.3107$\times 10^{5}$	5.0444$\times 10^{7}$	1.6465$\times 10^{1}$	1.3571$\times 10^{1}$	1.1523$\times 10^{2}$	7.5986	3.0053
		Std.	4.3072$\times 10^{2}$	9.9398$\times 10^{2}$	5.0155$\times 10^{6}$	1.5189$\times 10^{3}$	2.0091$\times 10^{4}$	3.2012$\times 10^{7}$	3.7228	1.2153	1.2196$\times 10^{1}$	7.5103$\times 10^{-1}$	7.2406$\times 10^{-1}$
	μ-DE	Min	4.7665$\times 10^{-1}$	1.2376$\times 10^{3}$	1.3169$\times 10^{3}$	1.9680$\times 10^{3}$	4.7300$\times 10^{4}$	6.3053$\times 10^{2}$	7.0966$\times 10^{-1}$	1.4194	5.3112$\times 10^{1}$	6.3199$\times 10^{-1}$	1.5434
		Max	3.1764$\times 10^{1}$	6.9069$\times 10^{3}$	1.5695$\times 10^{6}$	9.3974$\times 10^{3}$	1.4400$\times 10^{5}$	2.6189$\times 10^{4}$	1.4313	4.2528	6.9285$\times 10^{1}$	1.9982	4.9307
		Mean	7.1612	3.0649$\times 10^{3}$	6.9584$\times 10^{4}$	4.3416$\times 10^{3}$	9.5832$\times 10^{4}$	9.5438$\times 10^{3}$	9.7513$\times 10^{-1}$	2.4873	5.9932$\times 10^{1}$	1.2502	3.0597
		Std.	7.5619	1.3315$\times 10^{3}$	3.0620$\times 10^{5}$	1.7733$\times 10^{3}$	2.4355$\times 10^{4}$	7.9790$\times 10^{3}$	1.4832$\times 10^{-1}$	5.6313$\times 10^{-1}$	4.2171	3.7239$\times 10^{-1}$	8.8431$\times 10^{-1}$
	μ-DE-ERM	Min	5.3322$\times 10^{-1}$	6.3635$\times 10^{2}$	4.7781$\times 10^{2}$	1.6590$\times 10^{3}$	4.5700$\times 10^{4}$	1.3816$\times 10^{4}$	4.8680$\times 10^{-1}$	1.1239	5.2671$\times 10^{1}$	3.8493$\times 10^{-1}$	1.2788
		Max	7.4827$\times 10^{1}$	6.2309$\times 10^{3}$	2.9902$\times 10^{4}$	6.4038$\times 10^{3}$	1.7400$\times 10^{5}$	7.2442$\times 10^{9}$	1.0793	4.9250	6.7628$\times 10^{1}$	2.0128	4.7291
		Mean	5.9119	2.8106$\times 10^{3}$	6.0375$\times 10^{3}$	3.9850$\times 10^{3}$	1.0618$\times 10^{5}$	4.8361$\times 10^{8}$	8.5335$\times 10^{-1}$	2.3961	5.7127$\times 10^{1}$	9.3808$\times 10^{-1}$	2.8192
		Std.	1.4201$\times 10^{1}$	1.3761$\times 10^{3}$	5.7939$\times 10^{3}$	1.0291$\times 10^{3}$	2.8075$\times 10^{4}$	1.6645$\times 10^{9}$	1.2782$\times 10^{-1}$	8.3743$\times 10^{-1}$	3.7093	3.4194$\times 10^{-1}$	6.9376$\times 10^{-1}$
1$\times 10^{4}$	DE	Min	3.4300$\times 10^{-5}$	4.8700$\times 10^{2}$	1.4900$\times 10^{-1}$	5.9600$\times 10^{2}$	4.1400$\times 10^{4}$	2.4300$\times 10^{1}$	8.0500$\times 10^{-2}$	4.0100$\times 10^{-3}$	4.9700$\times 10^{1}$	1.8600$\times 10^{-2}$	4.5600$\times 10^{-1}$
		Max	4.4498$\times 10^{-4}$	1.2465$\times 10^{3}$	8.1222$\times 10^{-1}$	2.3151$\times 10^{3}$	8.8245$\times 10^{4}$	2.4549$\times 10^{2}$	2.5323$\times 10^{-1}$	8.5644$\times 10^{-3}$	4.9800$\times 10^{1}$	5.2567$\times 10^{-2}$	2.4412
		Mean	1.7725$\times 10^{-4}$	8.3623$\times 10^{2}$	3.7074$\times 10^{-1}$	1.3794$\times 10^{3}$	6.5638$\times 10^{4}$	1.1543$\times 10^{2}$	1.5877$\times 10^{-1}$	6.0944$\times 10^{-3}$	4.9751$\times 10^{1}$	3.5441$\times 10^{-2}$	1.5862
		Std.	1.0529$\times 10^{-4}$	2.2898$\times 10^{2}$	1.7374$\times 10^{-1}$	4.5977$\times 10^{2}$	1.2669$\times 10^{4}$	6.1691$\times 10^{1}$	5.1115$\times 10^{-2}$	1.4943$\times 10^{-3}$	4.4747$\times 10^{-2}$	7.9903$\times 10^{-3}$	5.3541$\times 10^{-1}$
	μ-DE	Min	7.4900$\times 10^{-30}$	1.5500$\times 10^{1}$	1.1800$\times 10^{-26}$	5.9500$\times 10^{1}$	2.1600$\times 10^{4}$	7.0900$\times 10^{-3}$	4.1200$\times 10^{-3}$	7.5500$\times 10^{-15}$	4.9700$\times 10^{1}$	0.0000	4.2000$\times 10^{-1}$
		Max	6.1365$\times 10^{-1}$	2.8232$\times 10^{2}$	5.8510$\times 10^{2}$	1.0005$\times 10^{3}$	5.6651$\times 10^{4}$	1.1849$\times 10^{2}$	7.9273$\times 10^{-2}$	1.6490	6.0692$\times 10^{1}$	4.5929$\times 10^{-1}$	2.4287
		Mean	2.5402$\times 10^{-2}$	8.7685$\times 10^{1}$	2.3421$\times 10^{1}$	4.5013$\times 10^{2}$	3.7459$\times 10^{4}$	1.8438$\times 10^{1}$	3.1591$\times 10^{-2}$	2.9704$\times 10^{-1}$	5.1221$\times 10^{1}$	4.0053$\times 10^{-2}$	1.5022
		Std.	1.2011$\times 10^{-1}$	6.5366$\times 10^{1}$	1.1465$\times 10^{2}$	2.5826$\times 10^{2}$	1.0697$\times 10^{4}$	2.8725$\times 10^{1}$	1.8098$\times 10^{-2}$	5.5969$\times 10^{-1}$	2.1212	9.3720$\times 10^{-2}$	4.4952$\times 10^{-1}$
	μ-DE-ERM	Min	2.4700$\times 10^{-31}$	2.3600	3.8100$\times 10^{-28}$	1.4200$\times 10^{1}$	2.6900$\times 10^{4}$	1.8700$\times 10^{-1}$	0.0000	4.0000$\times 10^{-15}$	4.9700$\times 10^{1}$	0.0000	1.1400
		Max	7.3037	9.5290$\times 10^{1}$	5.3315$\times 10^{3}$	5.5331$\times 10^{2}$	8.5402$\times 10^{4}$	6.2850$\times 10^{5}$	5.7068$\times 10^{-1}$	2.0559	7.7606$\times 10^{1}$	1.6091	2.4023
		Mean	3.9195$\times 10^{-1}$	1.8922$\times 10^{1}$	2.1465$\times 10^{2}$	2.1288$\times 10^{2}$	5.8078$\times 10^{4}$	2.5228$\times 10^{4}$	5.7840$\times 10^{-2}$	6.4234$\times 10^{-1}$	5.5322$\times 10^{1}$	1.8898$\times 10^{-1}$	1.7631
		Std.	1.4475	2.0798$\times 10^{1}$	1.0445$\times 10^{3}$	1.4733$\times 10^{2}$	1.6789$\times 10^{4}$	1.2314$\times 10^{5}$	1.2467$\times 10^{-1}$	7.2692$\times 10^{-1}$	7.7472	4.3263$\times 10^{-1}$	3.5940$\times 10^{-1}$
1$\times 10^{5}$	DE	Min	0.0000	2.7300$\times 10^{-3}$	0.0000	2.1900	9.5900$\times 10^{3}$	4.2200$\times 10^{-2}$	0.0000	4.0000$\times 10^{-15}$	4.9700$\times 10^{1}$	0.0000	4.1500$\times 10^{-1}$
		Max	3.1600$\times 10^{-30}$	9.3200$\times 10^{-2}$	0.0000	1.6200$\times 10^{1}$	5.0500$\times 10^{4}$	5.4500	0.0000	4.0000$\times 10^{-15}$	4.9700$\times 10^{1}$	0.0000	1.5300
		Mean	1.2837$\times 10^{-31}$	2.8105$\times 10^{-2}$	0.0000	7.2100	2.6092$\times 10^{4}$	9.5740$\times 10^{-1}$	0.0000	4.0000$\times 10^{-15}$	4.9700$\times 10^{1}$	0.0000	9.8676$\times 10^{-1}$
		Std.	6.1890$\times 10^{-31}$	2.0323$\times 10^{-2}$	0.0000	3.5283	9.3092$\times 10^{3}$	1.3015	0.0000	1.5777$\times 10^{-30}$	1.4211$\times 10^{-14}$	0.0000	2.6141$\times 10^{-1}$
	μ-DE	Min	0.0000	8.9600$\times 10^{-15}$	0.0000	4.5300$\times 10^{-7}$	2.4400$\times 10^{1}$	2.8300$\times 10^{-3}$	0.0000	4.0000$\times 10^{-15}$	4.9700$\times 10^{1}$	0.0000	3.1600$\times 10^{-1}$
		Max	4.4100$\times 10^{-4}$	1.0300$\times 10^{-3}$	6.6100$\times 10^{-2}$	2.2200	1.1300$\times 10^{4}$	5.9900$\times 10^{1}$	6.1800$\times 10^{-2}$	9.9400$\times 10^{-2}$	4.9800$\times 10^{1}$	6.7000$\times 10^{-2}$	1.3500
		Mean	1.7640$\times 10^{-5}$	4.1210$\times 10^{-5}$	2.6440$\times 10^{-3}$	1.1969$\times 10^{-1}$	2.8261$\times 10^{3}$	8.7498	2.5932$\times 10^{-2}$	3.9875$\times 10^{-3}$	4.9704$\times 10^{1}$	1.1997$\times 10^{-2}$	9.5124$\times 10^{-1}$
		Std.	8.6418$\times 10^{-5}$	2.0184$\times 10^{-4}$	1.2953$\times 10^{-2}$	4.3502$\times 10^{-1}$	3.6762$\times 10^{3}$	1.5577$\times 10^{1}$	1.4725$\times 10^{-2}$	1.9476$\times 10^{-2}$	1.9596$\times 10^{-2}$	2.1969$\times 10^{-2}$	2.8019$\times 10^{-1}$
	μ-DE-ERM	Min	9.8600$\times 10^{-31}$	1.4600$\times 10^{-28}$	0.0000	4.5600$\times 10^{-11}$	4.7100$\times 10^{3}$	5.0400$\times 10^{-2}$	0.0000	4.0000$\times 10^{-15}$	4.9700$\times 10^{1}$	0.0000	1.4400$\times 10^{-1}$
		Max	6.4400$\times 10^{-6}$	9.3100$\times 10^{-8}$	7.4000$\times 10^{3}$	8.4700$\times 10^{-5}$	6.3100$\times 10^{4}$	2.8800$\times 10^{7}$	4.8400$\times 10^{-1}$	4.5400	6.5700$\times 10^{1}$	1.6600	1.2700
		Mean	5.3240$\times 10^{-7}$	3.7240$\times 10^{-9}$	2.9601$\times 10^{2}$	3.6274$\times 10^{-6}$	2.3221$\times 10^{4}$	1.1522$\times 10^{6}$	5.0694$\times 10^{-2}$	3.9152$\times 10^{-1}$	5.4752$\times 10^{1}$	1.6547$\times 10^{-1}$	8.5560$\times 10^{-1}$
		Std.	1.6703$\times 10^{-6}$	1.8244$\times 10^{-8}$	1.4501$\times 10^{3}$	1.6565$\times 10^{-5}$	1.4997$\times 10^{4}$	5.6436$\times 10^{6}$	1.0589$\times 10^{-1}$	9.4743$\times 10^{-1}$	4.6625	3.4573$\times 10^{-1}$	2.6636$\times 10^{-1}$

**Table 5:** Results obtained with DE, μ-DE, and μ-DE-ERM over the shifted CEC 2005 benchmark problems after 25 independent runs, considering evaluation budgets of 1$\times 10^{3}$, 1$\times 10^{4}$, and 1$\times 10^{5}$.

FE	Algorithm	Index	Shifted $f_{1}$	Shifted $f_{2}$	Shifted $f_{3}$	Shifted $f_{4}$	Shifted $f_{5}$	Shifted $f_{6}$	Shifted $f_{7}$	Shifted $f_{8}$	Shifted $f_{9}$	Shifted $f_{10}$	Shifted $f_{11}$
1$\times 10^{3}$	DE	Min	3.4915$\times 10^{1}$	−2.5000$\times 10^{3}$	2.3600$\times 10^{6}$	4.0800$\times 10^{2}$	8.9400$\times 10^{4}$	8.4900$\times 10^{6}$	−1.7382$\times 10^{2}$	−1.2976$\times 10^{2}$	−2.3812$\times 10^{2}$	9.5902$\times 10^{1}$	−4.5803$\times 10^{2}$
		Max	1.7700$\times 10^{3}$	4.4700$\times 10^{3}$	2.6300$\times 10^{7}$	2.4600$\times 10^{3}$	1.5200$\times 10^{5}$	1.7800$\times 10^{8}$	−1.5545$\times 10^{2}$	−1.2427$\times 10^{2}$	−1.9769$\times 10^{2}$	9.9764$\times 10^{1}$	−4.5543$\times 10^{2}$
		Mean	8.9502$\times 10^{2}$	8.9767$\times 10^{2}$	1.0283$\times 10^{7}$	9.1048$\times 10^{2}$	1.2609$\times 10^{5}$	4.1612$\times 10^{7}$	−1.6641$\times 10^{2}$	−1.2672$\times 10^{2}$	−2.1212$\times 10^{2}$	9.8185$\times 10^{1}$	−4.5709$\times 10^{2}$
		Std.	4.5356$\times 10^{2}$	1.6983$\times 10^{3}$	5.6135$\times 10^{6}$	4.9555$\times 10^{2}$	1.6589$\times 10^{4}$	3.8429$\times 10^{7}$	4.5395	1.1527	8.1977	9.9945$\times 10^{-1}$	6.1416$\times 10^{-1}$
	μ-DE	Min	−4.4939$\times 10^{2}$	−3.2453$\times 10^{3}$	1.1784$\times 10^{3}$	9.6503$\times 10^{2}$	6.1200$\times 10^{4}$	9.1808$\times 10^{2}$	−1.7918$\times 10^{2}$	−1.3954$\times 10^{2}$	−2.7748$\times 10^{2}$	9.0471$\times 10^{1}$	−4.5854$\times 10^{2}$
		Max	−4.2774$\times 10^{2}$	1.2352$\times 10^{3}$	1.3998$\times 10^{5}$	7.9689$\times 10^{3}$	1.5800$\times 10^{5}$	1.2740$\times 10^{5}$	−1.7883$\times 10^{2}$	−1.3613$\times 10^{2}$	−2.5892$\times 10^{2}$	9.2153$\times 10^{1}$	−4.5488$\times 10^{2}$
		Mean	−4.4363$\times 10^{2}$	−1.3752$\times 10^{3}$	1.7290$\times 10^{4}$	3.8248$\times 10^{3}$	1.0924$\times 10^{5}$	1.6498$\times 10^{4}$	−1.7899$\times 10^{2}$	−1.3749$\times 10^{2}$	−2.7097$\times 10^{2}$	9.1240$\times 10^{1}$	−4.5714$\times 10^{2}$
		Std.	6.1889	1.2347$\times 10^{3}$	2.9235$\times 10^{4}$	1.7747$\times 10^{3}$	2.5240$\times 10^{4}$	2.9104$\times 10^{4}$	9.4390×10^−2^	7.4170$\times 10^{-1}$	4.9173	4.2090$\times 10^{-1}$	8.2506$\times 10^{-1}$
	μ-DE-ERM	Min	−4.5000$\times 10^{2}$	−4.3683$\times 10^{3}$	−1.9476$\times 10^{2}$	2.4589$\times 10^{2}$	5.5800$\times 10^{4}$	6.0775$\times 10^{2}$	−1.7951$\times 10^{2}$	−1.3924$\times 10^{2}$	−2.7829$\times 10^{2}$	9.0513$\times 10^{1}$	−4.5915$\times 10^{2}$
		Max	−4.5000$\times 10^{2}$	−2.2298$\times 10^{3}$	4.5714$\times 10^{6}$	6.8004$\times 10^{3}$	1.4400$\times 10^{5}$	1.5900$\times 10^{7}$	−1.7893$\times 10^{2}$	−1.3533$\times 10^{2}$	−2.6526$\times 10^{2}$	9.1709$\times 10^{1}$	−4.5564$\times 10^{2}$
		Mean	−4.5000$\times 10^{2}$	−3.6358$\times 10^{3}$	1.8973$\times 10^{5}$	3.6577$\times 10^{3}$	1.0776$\times 10^{5}$	6.5019$\times 10^{5}$	−1.7912$\times 10^{2}$	−1.3788$\times 10^{2}$	−2.7400$\times 10^{2}$	9.1006$\times 10^{1}$	−4.5724$\times 10^{2}$
		Std.	1.3248$\times 10^{-5}$	5.4395$\times 10^{2}$	8.9442$\times 10^{5}$	1.7741$\times 10^{3}$	2.3266$\times 10^{4}$	3.1131$\times 10^{6}$	1.5308$\times 10^{-1}$	9.6285$\times 10^{-1}$	2.9752	3.4057$\times 10^{-1}$	9.2188$\times 10^{-1}$
1$\times 10^{4}$	DE	Min	−4.5000$\times 10^{2}$	−4.2300$\times 10^{3}$	−4.5000$\times 10^{2}$	−4.4800$\times 10^{2}$	3.6200$\times 10^{4}$	4.1700$\times 10^{2}$	−1.8000$\times 10^{2}$	−1.4000$\times 10^{2}$	−2.8025$\times 10^{2}$	9.0000$\times 10^{1}$	−4.5900$\times 10^{2}$
		Max	−4.5000$\times 10^{2}$	−2.8900$\times 10^{3}$	−4.4897$\times 10^{2}$	−4.3500$\times 10^{2}$	8.0700$\times 10^{4}$	5.4549$\times 10^{2}$	−1.7977$\times 10^{2}$	−1.3999$\times 10^{2}$	−2.8000$\times 10^{2}$	9.0049$\times 10^{1}$	−4.5791$\times 10^{2}$
		Mean	−4.5000$\times 10^{2}$	−3.6032$\times 10^{3}$	−4.4986$\times 10^{2}$	−4.4276$\times 10^{2}$	5.6544$\times 10^{4}$	4.8095$\times 10^{2}$	−1.7996$\times 10^{2}$	−1.4000$\times 10^{2}$	−2.8005$\times 10^{2}$	9.0009$\times 10^{1}$	−4.5836$\times 10^{2}$
		Std.	1.6733$\times 10^{-4}$	3.1018$\times 10^{2}$	2.9925$\times 10^{-1}$	3.7017	1.3167$\times 10^{4}$	3.9838$\times 10^{1}$	8.2053$\times 10^{-2}$	4.4680$\times 10^{-3}$	9.8834$\times 10^{-2}$	1.8318$\times 10^{-2}$	4.7939$\times 10^{-1}$
	μ-DE	Min	−4.5000$\times 10^{2}$	−4.4900$\times 10^{3}$	−4.5000$\times 10^{2}$	−3.8800$\times 10^{2}$	1.9200$\times 10^{4}$	3.9000$\times 10^{2}$	−1.8000$\times 10^{2}$	−1.4000$\times 10^{2}$	−2.8000$\times 10^{2}$	9.0000$\times 10^{1}$	−4.6000$\times 10^{2}$
		Max	−4.4990$\times 10^{2}$	−4.2052$\times 10^{3}$	−2.6312$\times 10^{2}$	6.9979$\times 10^{2}$	6.2893$\times 10^{4}$	1.8156$\times 10^{3}$	−1.7993$\times 10^{2}$	−1.3883$\times 10^{2}$	−2.7331$\times 10^{2}$	9.0264$\times 10^{1}$	−4.5753$\times 10^{2}$
		Mean	−4.4999$\times 10^{2}$	−4.4131$\times 10^{3}$	−4.3823$\times 10^{2}$	−2.3074$\times 10^{1}$	4.0482$\times 10^{4}$	4.5544$\times 10^{2}$	−1.7999$\times 10^{2}$	−1.3993$\times 10^{2}$	−2.7892$\times 10^{2}$	9.0038$\times 10^{1}$	−4.5828$\times 10^{2}$
		Std.	2.5663 $\times 10^{-2}$	7.0336$\times 10^{1}$	4.1386$\times 10^{1}$	2.6424$\times 10^{2}$	1.1023$\times 10^{4}$	2.7781$\times 10^{2}$	2.3482$\times 10^{-2}$	2.3754$\times 10^{-1}$	1.4114	7.6291$\times 10^{-2}$	5.8008$\times 10^{-1}$
	μ-DE-ERM	Min	−4.5000$\times 10^{2}$	−4.5000$\times 10^{3}$	−4.5000$\times 10^{2}$	−4.3900$\times 10^{2}$	1.9500$\times 10^{4}$	3.9000$\times 10^{2}$	−1.8000$\times 10^{2}$	−1.4000$\times 10^{2}$	−2.8000$\times 10^{2}$	9.0000$\times 10^{1}$	−4.5900$\times 10^{2}$
		Max	−4.3200$\times 10^{2}$	−4.1150$\times 10^{3}$	−3.7468$\times 10^{2}$	7.4304$\times 10^{2}$	9.0366$\times 10^{4}$	7.9576$\times 10^{2}$	−1.7978$\times 10^{2}$	−1.3570$\times 10^{2}$	−2.6234$\times 10^{2}$	9.0997$\times 10^{1}$	−4.5743$\times 10^{2}$
		Mean	−4.4903$\times 10^{2}$	−4.4846$\times 10^{3}$	−4.4696$\times 10^{2}$	−9.6072$\times 10^{1}$	5.4142$\times 10^{4}$	4.2598$\times 10^{2}$	−1.7997$\times 10^{2}$	−1.3917$\times 10^{2}$	−2.7650$\times 10^{2}$	9.0127$\times 10^{1}$	−4.5813$\times 10^{2}$
		Std.	3.6554	7.5448$\times 10^{1}$	1.4755$\times 10^{1}$	3.7748$\times 10^{2}$	2.1825$\times 10^{4}$	8.0311$\times 10^{1}$	5.6592$\times 10^{-2}$	1.2193	4.0732	2.5214$\times 10^{-1}$	4.5483$\times 10^{-1}$
1$\times 10^{5}$	DE	Min	−4.5000$\times 10^{2}$	−4.5000$\times 10^{3}$	−4.5000$\times 10^{2}$	−4.4900$\times 10^{2}$	5.3900$\times 10^{3}$	3.9000$\times 10^{2}$	−1.8000$\times 10^{2}$	−1.4000$\times 10^{2}$	−2.8000$\times 10^{2}$	9.0000$\times 10^{1}$	−4.5900$\times 10^{2}$
		Max	−4.5000$\times 10^{2}$	−4.5000$\times 10^{3}$	−4.5000$\times 10^{2}$	−4.3500$\times 10^{2}$	4.5000$\times 10^{4}$	3.9300$\times 10^{2}$	−1.8000$\times 10^{2}$	−1.4000$\times 10^{2}$	−2.8000$\times 10^{2}$	9.0000$\times 10^{1}$	−4.5900$\times 10^{2}$
		Mean	−4.5000$\times 10^{2}$	−4.5000$\times 10^{3}$	−4.5000$\times 10^{2}$	−4.4344$\times 10^{2}$	2.2604$\times 10^{4}$	3.9100$\times 10^{2}$	−1.8000$\times 10^{2}$	−1.4000$\times 10^{2}$	−2.8000$\times 10^{2}$	9.0000$\times 10^{1}$	−4.5900$\times 10^{2}$
		Std.	0.0000	0.0000	0.0000	3.9606	9.4036$\times 10^{3}$	7.4833$\times 10^{-1}$	0.0000	0.0000	0.0000	0.0000	0.0000
	μ-DE	Min	−4.5000$\times 10^{2}$	−4.5000$\times 10^{3}$	−4.5000$\times 10^{2}$	−4.5000$\times 10^{2}$	−3.0900$\times 10^{2}$	3.9000$\times 10^{2}$	−1.8000$\times 10^{2}$	−1.4000$\times 10^{2}$	−2.8000$\times 10^{2}$	9.0000$\times 10^{1}$	−4.6000$\times 10^{2}$
		Max	−4.5000$\times 10^{2}$	−4.5000$\times 10^{3}$	1.0900$\times 10^{2}$	−4.4800$\times 10^{2}$	2.0400$\times 10^{4}$	4.7200$\times 10^{2}$	−1.8000$\times 10^{2}$	−1.4000$\times 10^{2}$	−2.8000$\times 10^{2}$	9.0100$\times 10^{1}$	−4.5900$\times 10^{2}$
		Mean	−4.5000$\times 10^{2}$	−4.5000$\times 10^{3}$	−4.2764$\times 10^{2}$	−4.4992$\times 10^{2}$	4.2595$\times 10^{3}$	3.9768$\times 10^{2}$	−1.8000$\times 10^{2}$	−1.4000$\times 10^{2}$	−2.8000$\times 10^{2}$	9.0008$\times 10^{1}$	−4.5908$\times 10^{2}$
		Std.	0.0000	0.0000	1.0954$\times 10^{2}$	3.9192$\times 10^{-1}$	6.1002$\times 10^{3}$	1.8814$\times 10^{1}$	0.0000	0.0000	0.0000	2.7129$\times 10^{-2}$	2.7129$\times 10^{-1}$
	μ-DE-ERM	Min	−4.5000$\times 10^{2}$	−4.5000$\times 10^{3}$	−4.5000$\times 10^{2}$	−4.5000$\times 10^{2}$	8.1200$\times 10^{3}$	3.9000$\times 10^{2}$	−1.8000$\times 10^{2}$	−1.4000$\times 10^{2}$	−2.8000$\times 10^{2}$	9.0000$\times 10^{1}$	−4.5900$\times 10^{2}$
		Max	−4.2000$\times 10^{2}$	−4.5000$\times 10^{3}$	7.3700$\times 10^{4}$	−4.5000$\times 10^{2}$	7.1100$\times 10^{4}$	4.3900$\times 10^{2}$	−1.8000$\times 10^{2}$	−1.3600$\times 10^{2}$	−2.6300$\times 10^{2}$	9.0900$\times 10^{1}$	−4.5900$\times 10^{2}$
		Mean	−4.4868$\times 10^{2}$	−4.5000$\times 10^{3}$	2.5160$\times 10^{3}$	−4.5000$\times 10^{2}$	2.5834$\times 10^{4}$	4.0080$\times 10^{2}$	−1.8000$\times 10^{2}$	−1.3916$\times 10^{2}$	−2.7672$\times 10^{2}$	9.0136$\times 10^{1}$	−4.5900$\times 10^{2}$
		Std.	5.8837	0.0000	1.4530$\times 10^{4}$	0.0000	1.4277$\times 10^{4}$	1.4786$\times 10^{1}$	0.0000	1.2225	4.1619	2.2606$\times 10^{-1}$	0.0000

**Table 6:** Results obtained with DE, μ-DE, and μ-DE-ERM over the rotated CEC 2005 benchmark problems after 25 independent runs, considering evaluation budgets of 1$\times 10^{3}$, 1$\times 10^{4}$, and 1$\times 10^{5}$.

FE	Algorithm	Index	Rotated $f_{1}$	Rotated $f_{2}$	Rotated $f_{3}$	Rotated $f_{4}$	Rotated $f_{5}$	Rotated $f_{6}$	Rotated $f_{7}$	Rotated $f_{8}$	Rotated $f_{9}$	Rotated $f_{10}$	Rotated $f_{11}$
1$\times 10^{3}$	DE	Min	2.0441$\times 10^{1}$	6.0692$\times 10^{1}$	1.9200$\times 10^{4}$	8.4057$\times 10^{1}$	5.1000$\times 10^{3}$	1.9200$\times 10^{4}$	9.7100$\times 10^{-1}$	4.4498	1.1638$\times 10^{2}$	9.0469	1.4420
		Max	2.1854$\times 10^{2}$	3.5278$\times 10^{2}$	8.4200$\times 10^{5}$	2.6542$\times 10^{2}$	3.0000$\times 10^{4}$	8.4200$\times 10^{5}$	1.3300	8.8627	2.0080$\times 10^{2}$	1.2321$\times 10^{1}$	4.3992
		Mean	9.1041$\times 10^{1}$	1.7233$\times 10^{2}$	3.0638$\times 10^{5}$	1.6697$\times 10^{2}$	1.5236$\times 10^{4}$	3.0638$\times 10^{5}$	1.1066	6.0861	1.5939$\times 10^{2}$	1.0267$\times 10^{1}$	2.8241
		Std.	4.9760$\times 10^{1}$	8.1022$\times 10^{1}$	2.1911$\times 10^{5}$	5.2070$\times 10^{1}$	5.5456$\times 10^{3}$	2.1911$\times 10^{5}$	8.4601$\times 10^{-2}$	9.8381$\times 10^{-1}$	2.4216$\times 10^{1}$	8.5327$\times 10^{-1}$	7.0876$\times 10^{-1}$
	μ-DE	Min	2.7000$\times 10^{-2}$	5.0393$\times 10^{1}$	1.0622$\times 10^{2}$	1.0974$\times 10^{2}$	9.5000$\times 10^{3}$	2.8861$\times 10^{2}$	5.1730$\times 10^{-1}$	2.1950$\times 10^{-1}$	5.5348$\times 10^{1}$	9.0660$\times 10^{-1}$	1.0706
		Max	1.0354	4.5575$\times 10^{2}$	3.0200$\times 10^{4}$	6.5303$\times 10^{2}$	5.9400$\times 10^{4}$	6.1600$\times 10^{3}$	9.9020$\times 10^{-1}$	3.9730	1.1893$\times 10^{2}$	6.8559	3.7679
		Mean	2.5670$\times 10^{-1}$	2.5096$\times 10^{2}$	3.4385$\times 10^{3}$	2.5990$\times 10^{2}$	3.3152$\times 10^{4}$	1.5818$\times 10^{3}$	7.5704$\times 10^{-1}$	1.3419	7.1173$\times 10^{1}$	2.5907	2.5738
		Std.	2.5790$\times 10^{-1}$	1.1313$\times 10^{2}$	7.0074$\times 10^{3}$	1.3379$\times 10^{2}$	1.1160$\times 10^{4}$	1.5095$\times 10^{3}$	1.3599$\times 10^{-1}$	9.5201$\times 10^{-1}$	1.4601$\times 10^{1}$	1.4445	6.8342$\times 10^{-1}$
	μ-DE-ERM	Min	2.3978$\times 10^{-2}$	3.7044$\times 10^{1}$	7.2552$\times 10^{1}$	5.2233$\times 10^{1}$	1.1600$\times 10^{4}$	8.9462$\times 10^{1}$	2.3378$\times 10^{-1}$	2.6333$\times 10^{-1}$	5.4571$\times 10^{1}$	6.5837$\times 10^{-1}$	1.3597
		Max	1.2538	4.9393$\times 10^{2}$	2.1574$\times 10^{4}$	6.5743$\times 10^{2}$	4.4200$\times 10^{4}$	8.6982$\times 10^{3}$	7.2974$\times 10^{-1}$	7.7152	1.1831$\times 10^{2}$	7.6052	4.5186
		Mean	2.4701$\times 10^{-1}$	1.8046$\times 10^{2}$	2.1550$\times 10^{3}$	2.4289$\times 10^{2}$	2.8096$\times 10^{4}$	9.8717$\times 10^{2}$	5.1251$\times 10^{-1}$	1.2606	7.4093$\times 10^{1}$	4.1198	2.8673
		Std.	3.1155$\times 10^{-1}$	1.0368$\times 10^{2}$	5.6070$\times 10^{3}$	1.5040$\times 10^{2}$	7.9231$\times 10^{3}$	1.6996$\times 10^{3}$	1.3300$\times 10^{-1}$	1.4512	1.8826$\times 10^{1}$	1.8956	9.0325$\times 10^{-1}$
1$\times 10^{4}$	DE	Min	0.0000	2.9400	4.7000$\times 10^{-3}$	1.0600$\times 10^{1}$	3.0600$\times 10^{3}$	2.0400$\times 10^{3}$	2.3000$\times 10^{-2}$	8.0000$\times 10^{-4}$	4.9700$\times 10^{1}$	2.5100$\times 10^{-2}$	6.5100$\times 10^{-1}$
		Max	3.8783	5.5289$\times 10^{1}$	7.8300$\times 10^{5}$	2.2413$\times 10^{2}$	1.1800$\times 10^{4}$	7.5800$\times 10^{3}$	1.8200$\times 10^{-1}$	2.7000$\times 10^{-3}$	1.4227$\times 10^{2}$	4.5347	2.2936
		Mean	1.5524$\times 10^{-1}$	1.8016$\times 10^{1}$	3.4248$\times 10^{4}$	5.2953$\times 10^{1}$	6.7504$\times 10^{3}$	4.0916$\times 10^{3}$	9.6916$\times 10^{-2}$	1.5440$\times 10^{-3}$	6.1208$\times 10^{1}$	6.5300$\times 10^{-1}$	1.6189
		Std.	7.5997$\times 10^{-1}$	1.0520$\times 10^{1}$	1.5351$\times 10^{5}$	4.1867$\times 10^{1}$	2.5077$\times 10^{3}$	1.4243$\times 10^{3}$	3.6580$\times 10^{-2}$	4.4907$\times 10^{-4}$	2.0278$\times 10^{1}$	1.0480	3.9829$\times 10^{-1}$
	μ-DE	Min	9.8600$\times 10^{-31}$	3.1800	4.2100$\times 10^{-29}$	8.8600	5.8100$\times 10^{3}$	1.4600$\times 10^{-1}$	0.0000	7.5500$\times 10^{-15}$	4.9700$\times 10^{1}$	0.0000	1.1300
		Max	2.7600$\times 10^{-2}$	6.3630$\times 10^{1}$	6.3695$\times 10^{2}$	2.0361$\times 10^{2}$	2.7500$\times 10^{4}$	2.4427$\times 10^{1}$	1.3280$\times 10^{-1}$	1.1551	6.1693$\times 10^{1}$	2.6260$\times 10^{-1}$	2.3488
		Mean	1.1099$\times 10^{-3}$	2.1330$\times 10^{1}$	2.5578$\times 10^{1}$	5.8970$\times 10^{1}$	1.1864$\times 10^{4}$	5.1249	3.3012$\times 10^{-2}$	1.0498$\times 10^{-1}$	5.1157$\times 10^{1}$	3.7563$\times 10^{-2}$	1.7839
		Std.	5.4073$\times 10^{-3}$	1.8030$\times 10^{1}$	1.2480$\times 10^{2}$	5.0150$\times 10^{1}$	4.6029$\times 10^{3}$	5.5790	3.0933$\times 10^{-2}$	3.1174$\times 10^{-1}$	2.3645	6.5009$\times 10^{-2}$	3.1198$\times 10^{-1}$
	μ-DE-ERM	Min	0.0000	1.6900$\times 10^{-2}$	1.4600$\times 10^{-29}$	1.6500	5.2400$\times 10^{3}$	7.1500$\times 10^{-1}$	0.0000	4.0000$\times 10^{-15}$	4.9700$\times 10^{1}$	0.0000	7.3800$\times 10^{-1}$
		Max	4.2123$\times 10^{-4}$	3.5962	8.7824$\times 10^{3}$	8.2465$\times 10^{1}$	3.7142$\times 10^{4}$	2.0533$\times 10^{3}$	3.0154$\times 10^{-1}$	1.6747	1.3730$\times 10^{2}$	4.5000	2.4517
		Mean	1.6878$\times 10^{-5}$	9.7916$\times 10^{-1}$	3.5207$\times 10^{2}$	2.4562$\times 10^{1}$	1.8182$\times 10^{4}$	9.8127$\times 10^{1}$	3.8433$\times 10^{-2}$	3.8393$\times 10^{-1}$	6.8903$\times 10^{1}$	1.7618	1.5513
		Std.	8.2539$\times 10^{-5}$	9.8144$\times 10^{-1}$	1.7208$\times 10^{3}$	2.4017$\times 10^{1}$	8.0765$\times 10^{3}$	4.0020$\times 10^{2}$	5.8278$\times 10^{-2}$	6.2930$\times 10^{-1}$	2.3657$\times 10^{1}$	1.2629	3.9434$\times 10^{-1}$
1$\times 10^{5}$	DE	Min	0.0000	5.7400$\times 10^{-5}$	0.0000	2.2200$\times 10^{-2}$	1.3500$\times 10^{3}$	6.0800$\times 10^{2}$	0.0000	4.0000$\times 10^{-15}$	4.9700$\times 10^{1}$	0.0000	5.1900$\times 10^{-1}$
		Max	5.3900	2.3000$\times 10^{-3}$	2.4700$\times 10^{4}$	3.1900$\times 10^{-1}$	1.2600$\times 10^{4}$	4.3900$\times 10^{3}$	0.0000	4.0000$\times 10^{-15}$	2.2100$\times 10^{2}$	4.5800	1.3400
		Mean	2.2528$\times 10^{-1}$	5.4531$\times 10^{-4}$	1.2691$\times 10^{3}$	1.3046$\times 10^{-1}$	4.0544$\times 10^{3}$	1.7784$\times 10^{3}$	0.0000	4.0000$\times 10^{-15}$	7.8212$\times 10^{1}$	4.2320$\times 10^{-1}$	9.4160$\times 10^{-1}$
		Std.	1.0553	5.9397$\times 10^{-4}$	4.8752$\times 10^{3}$	8.3680$\times 10^{-2}$	2.8931$\times 10^{3}$	1.0565$\times 10^{3}$	0.0000	1.5777$\times 10^{-30}$	4.4753$\times 10^{1}$	1.0099	2.0858$\times 10^{-1}$
	μ-DE	Min	0.0000	4.2000$\times 10^{-13}$	0.0000	4.2900$\times 10^{-6}$	1.4400$\times 10^{1}$	7.0300$\times 10^{-12}$	0.0000	4.0000$\times 10^{-15}$	4.9700$\times 10^{1}$	0.0000	5.2100$\times 10^{-1}$
		Max	1.4000$\times 10^{-6}$	4.8800$\times 10^{-4}$	1.3700$\times 10^{-7}$	3.2800$\times 10^{1}$	7.8100$\times 10^{3}$	1.6600$\times 10^{1}$	7.8700$\times 10^{-2}$	2.7200$\times 10^{-2}$	4.9700$\times 10^{1}$	6.5800$\times 10^{-2}$	1.7700
		Mean	5.6000$\times 10^{-8}$	1.9584$\times 10^{-5}$	5.4800$\times 10^{-9}$	1.3196	1.3829$\times 10^{3}$	3.2421	2.1527$\times 10^{-2}$	1.6692$\times 10^{-3}$	4.9700$\times 10^{1}$	6.7780$\times 10^{-3}$	9.2716$\times 10^{-1}$
		Std.	2.7434$\times 10^{-7}$	9.5615$\times 10^{-5}$	2.6846$\times 10^{-8}$	6.4260	1.6467$\times 10^{3}$	4.5630	1.6915$\times 10^{-2}$	5.7810$\times 10^{-3}$	1.4211$\times 10^{-14}$	1.4184$\times 10^{-2}$	2.5493$\times 10^{-1}$
	μ-DE-ERM	Min	3.3500$\times 10^{-30}$	4.2600$\times 10^{-29}$	0.0000	4.6900$\times 10^{-13}$	1.1000$\times 10^{3}$	8.5700$\times 10^{-4}$	0.0000	4.0000$\times 10^{-15}$	4.9700$\times 10^{1}$	3.4100$\times 10^{-5}$	4.6900$\times 10^{-1}$
		Max	6.5400$\times 10^{-2}$	1.1000$\times 10^{-6}$	3.3800$\times 10^{-1}$	3.9800$\times 10^{-2}$	1.3900$\times 10^{4}$	2.8800$\times 10^{1}$	1.0600$\times 10^{-1}$	4.5400	1.9600$\times 10^{2}$	9.0800	1.2700
		Mean	2.6160$\times 10^{-3}$	4.4000$\times 10^{-8}$	2.0201$\times 10^{-2}$	1.5929$\times 10^{-3}$	5.5944$\times 10^{3}$	3.0969	2.6230$\times 10^{-2}$	6.4200$\times 10^{-1}$	9.0704$\times 10^{1}$	3.6288	9.1448$\times 10^{-1}$
		Std.	1.2816$\times 10^{-2}$	2.1556$\times 10^{-7}$	7.2283$\times 10^{-2}$	7.7990$\times 10^{-3}$	3.4876$\times 10^{3}$	6.3092	2.3804$\times 10^{-2}$	1.1641	4.3521$\times 10^{1}$	2.1710	2.2032$\times 10^{-1}$

**Table 7:** Best performance counts by algorithm based on the problem type and the FE for the CEC 2005 benchmark problems.

Problem Type	FE	Algorithm	Min	Max	Mean	Std.	Total
Base	1$\times 10^{3}$	DE	0	1	0	2	3
		μ-DE	2	5	2	3	12
		μ-DE-ERM	9	5	9	6	29
	1$\times 10^{4}$	DE	1	5	5	5	16
		μ-DE	5	3	4	3	15
		μ-DE-ERM	8	3	2	3	16
	1$\times 10^{5}$	DE	6	7	7	8	28
		μ-DE	8	1	1	1	11
		μ-DE-ERM	8	3	3	2	16
Shifted	1$\times 10^{3}$	DE	0	1	1	3	5
		μ-DE	2	3	2	4	11
		μ-DE-ERM	9	7	8	4	28
	1$\times 10^{4}$	DE	7	8	7	7	29
		μ-DE	8	3	2	3	16
		μ-DE-ERM	7	0	2	1	10
	1$\times 10^{5}$	DE	8	9	8	9	34
		μ-DE	11	7	7	6	31
		μ-DE-ERM	9	4	3	4	20
Rotated	1$\times 10^{3}$	DE	1	3	3	5	12
		μ-DE	2	5	3	5	15
		μ-DE-ERM	8	3	5	1	17
	1$\times 10^{4}$	DE	4	3	2	2	11
		μ-DE	4	5	5	6	20
		μ-DE-ERM	8	3	4	3	18
	1$\times 10^{5}$	DE	6	2	2	3	13
		μ-DE	8	6	5	6	25
		μ-DE-ERM	7	3	4	2	16

**Table 8:** Results of the Kruskal-Wallis tests for the CEC 2005 benchmark problems.

Problem Type	FE	Index	$f_{1}$	$f_{2}$	$f_{3}$	$f_{4}$	$f_{5}$	$f_{6}$	$f_{7}$	$f_{8}$	$f_{9}$	$f_{10}$	$f_{11}$
Base	1$\times 10^{3}$	*p*-value	8.8090$\times 10^{-12}$	1.7060$\times 10^{-6}$	1.4146$\times 10^{-11}$	1.9405$\times 10^{-6}$	2.6760$\times 10^{-5}$	3.5968$\times 10^{-13}$	3.9270$\times 10^{-12}$	1.8269$\times 10^{-11}$	4.8041$\times 10^{-12}$	2.4534$\times 10^{-12}$	6.5763$\times 10^{-1}$
		*H*	5.0910$\times 10^{1}$	2.6563$\times 10^{1}$	4.9963$\times 10^{1}$	2.6305$\times 10^{1}$	2.1057$\times 10^{1}$	5.7307$\times 10^{1}$	5.2526$\times 10^{1}$	4.9452$\times 10^{1}$	5.2123$\times 10^{1}$	5.3467$\times 10^{1}$	8.3823$\times 10^{-1}$
	1$\times 10^{4}$	*p*-value	5.5700$\times 10^{-6}$	8.9869$\times 10^{-14}$	2.3834$\times 10^{-8}$	3.6846$\times 10^{-12}$	8.5911$\times 10^{-8}$	3.4597$\times 10^{-7}$	8.2522$\times 10^{-10}$	3.2829$\times 10^{-1}$	6.1000$\times 10^{-8}$	1.3934$\times 10^{-3}$	1.4704$\times 10^{-1}$
		*H*	2.4196$\times 10^{1}$	6.0081$\times 10^{1}$	3.5104$\times 10^{1}$	5.2654$\times 10^{1}$	3.2540$\times 10^{1}$	2.9754$\times 10^{1}$	4.1831$\times 10^{1}$	2.2277	3.3225$\times 10^{1}$	1.3152$\times 10^{1}$	3.8342
	1$\times 10^{5}$	*p*-value	3.1692$\times 10^{-12}$	1.7431$\times 10^{-14}$	3.2651$\times 10^{-10}$	1.0141$\times 10^{-14}$	6.8476$\times 10^{-11}$	6.8843$\times 10^{-4}$	2.9550$\times 10^{-11}$	8.0423$\times 10^{-7}$	6.3854$\times 10^{-14}$	1.0726$\times 10^{-8}$	2.4811$\times 10^{-1}$
		*H*	5.2955$\times 10^{1}$	6.3361$\times 10^{1}$	4.3685$\times 10^{1}$	6.4444$\times 10^{1}$	4.6809$\times 10^{1}$	1.4562$\times 10^{1}$	4.8490$\times 10^{1}$	2.8067$\times 10^{1}$	6.0764$\times 10^{1}$	3.6701$\times 10^{1}$	2.7878
Shifted	1$\times 10^{3}$	*p*-value	3.2636$\times 10^{-15}$	9.0903$\times 10^{-13}$	1.8818$\times 10^{-11}$	1.3680$\times 10^{-9}$	9.9803$\times 10^{-3}$	2.7282$\times 10^{-11}$	2.4534$\times 10^{-12}$	8.2435$\times 10^{-12}$	6.2750$\times 10^{-12}$	8.1044$\times 10^{-12}$	9.5426$\times 10^{-1}$
		*H*	6.6712$\times 10^{1}$	5.5453$\times 10^{1}$	4.9392$\times 10^{1}$	4.0820$\times 10^{1}$	9.2143	4.8650$\times 10^{1}$	5.3467$\times 10^{1}$	5.1043$\times 10^{1}$	5.1589$\times 10^{1}$	5.1077$\times 10^{1}$	9.3642$\times 10^{-2}$
	1$\times 10^{4}$	*p*-value	9.5982$\times 10^{-1}$	1.1561$\times 10^{-14}$	9.8408$\times 10^{-1}$	1.1340$\times 10^{-11}$	9.8209$\times 10^{-4}$	2.1164$\times 10^{-8}$	9.1988$\times 10^{-1}$	1.8164$\times 10^{-2}$	1.6461$\times 10^{-10}$	3.0742$\times 10^{-1}$	1.2545$\times 10^{-1}$
		*H*	8.2025$\times 10^{-2}$	6.4182$\times 10^{1}$	3.2093$\times 10^{-2}$	5.0405$\times 10^{1}$	1.3852$\times 10^{1}$	3.5342$\times 10^{1}$	1.6702$\times 10^{-1}$	8.0166	4.5055$\times 10^{1}$	2.3591	4.1517
	1$\times 10^{5}$	*p*-value	1.3173$\times 10^{-1}$	1.0000	6.0228$\times 10^{-1}$	1.9190$\times 10^{-15}$	1.6085$\times 10^{-9}$	1.2806$\times 10^{-2}$	1.0000	3.2685$\times 10^{-6}$	4.6293$\times 10^{-12}$	1.7395$\times 10^{-4}$	1.3168$\times 10^{-1}$
		*H*	4.0541	0.0000	1.0141	6.7774$\times 10^{1}$	4.0496$\times 10^{1}$	8.7157	0.0000	2.5262$\times 10^{1}$	5.2197$\times 10^{1}$	1.7313$\times 10^{1}$	4.0548
Rotated	1$\times 10^{3}$	*p*-value	1.7033$\times 10^{-11}$	1.8685$\times 10^{-2}$	4.2990$\times 10^{-12}$	4.7502$\times 10^{-2}$	2.7958$\times 10^{-8}$	2.5853$\times 10^{-12}$	1.1587$\times 10^{-13}$	1.1443$\times 10^{-10}$	2.4095$\times 10^{-11}$	3.0890$\times 10^{-12}$	4.5577$\times 10^{-1}$
		*H*	4.9592$\times 10^{1}$	7.9601	5.2345$\times 10^{1}$	6.0940	3.4785$\times 10^{1}$	5.3362$\times 10^{1}$	5.9573$\times 10^{1}$	4.5782$\times 10^{1}$	4.8898$\times 10^{1}$	5.3006$\times 10^{1}$	1.5715
	1$\times 10^{4}$	*p*-value	2.4088$\times 10^{-8}$	2.8238$\times 10^{-11}$	4.4909$\times 10^{-7}$	3.8198$\times 10^{-4}$	7.3506$\times 10^{-9}$	8.0947$\times 10^{-12}$	5.5920$\times 10^{-8}$	3.3442$\times 10^{-3}$	7.8816$\times 10^{-5}$	1.6924$\times 10^{-6}$	8.5833$\times 10^{-2}$
		*H*	3.5083$\times 10^{1}$	4.8581$\times 10^{1}$	2.9232$\times 10^{1}$	1.5740$\times 10^{1}$	3.7457$\times 10^{1}$	5.1080$\times 10^{1}$	3.3399$\times 10^{1}$	1.1401$\times 10^{1}$	1.8897$\times 10^{1}$	2.6579$\times 10^{1}$	4.9107
	1$\times 10^{5}$	*p*-value	1.6913$\times 10^{-9}$	4.9713$\times 10^{-14}$	1.8941$\times 10^{-4}$	2.3554$\times 10^{-13}$	2.3074$\times 10^{-7}$	1.5877$\times 10^{-11}$	2.1424$\times 10^{-10}$	1.9558$\times 10^{-6}$	9.1284$\times 10^{-11}$	1.3470$\times 10^{-10}$	9.3640$\times 10^{-1}$
		*H*	4.0396$\times 10^{1}$	6.1265$\times 10^{1}$	1.7143$\times 10^{1}$	5.8154$\times 10^{1}$	3.0564$\times 10^{1}$	4.9732$\times 10^{1}$	4.4528$\times 10^{1}$	2.6289$\times 10^{1}$	4.6234$\times 10^{1}$	4.5456$\times 10^{1}$	1.3143$\times 10^{-1}$

**Table 9:** Results of the Mann-Whitney U tests for the CEC 2005 benchmark problems.

Problem Type	FE	Test	$f_{1}$	$f_{2}$	$f_{3}$	$f_{4}$	$f_{5}$	$f_{6}$	$f_{7}$	$f_{8}$	$f_{9}$	$f_{10}$	$f_{11}$
Base	1$\times 10^{3}$	DE vs. μ-DE	−4.2470$\times 10^{-9}$	−2.7698$\times 10^{-5}$	−4.2470$\times 10^{-9}$	−1.9241$\times 10^{-4}$	−3.7879$\times 10^{-5}$	−4.2470$\times 10^{-9}$	−4.2470$\times 10^{-9}$	−4.2470$\times 10^{-9}$	−4.2470$\times 10^{-9}$	−4.2470$\times 10^{-9}$	−
		DE vs. μ-DE-ERM	−4.2470$\times 10^{-9}$	−2.2178$\times 10^{-5}$	−4.2470$\times 10^{-9}$	−3.0325$\times 10^{-6}$	−2.9192$\times 10^{-3}$	−3.8768$\times 10^{-6}$	−4.2470$\times 10^{-9}$	−4.2470$\times 10^{-9}$	−4.2470$\times 10^{-9}$	−4.2470$\times 10^{-9}$	−
		μ-DE vs. μ-DE-ERM	≈6.2508$\times 10^{-2}$	≈4.6093$\times 10^{-1}$	≈1.9360$\times 10^{-1}$	≈7.8590$\times 10^{-1}$	≈1.5379$\times 10^{-1}$	+1.4759$\times 10^{-8}$	−7.8561$\times 10^{-3}$	≈6.2763$\times 10^{-1}$	−1.3007$\times 10^{-2}$	−2.4712$\times 10^{-3}$	−
	1$\times 10^{4}$	DE vs. μ-DE	−2.9073$\times 10^{-5}$	−4.2431$\times 10^{-9}$	−5.3861$\times 10^{-7}$	−2.3327$\times 10^{-8}$	−2.0643$\times 10^{-7}$	−1.6631$\times 10^{-7}$	−4.2355$\times 10^{-9}$	−	+1.4855$\times 10^{-5}$	−2.0490$\times 10^{-3}$	−
		DE vs. μ-DE-ERM	−1.1810$\times 10^{-4}$	−4.2431$\times 10^{-9}$	−1.6588$\times 10^{-6}$	−4.2470$\times 10^{-9}$	≈1.0108$\times 10^{-1}$	−2.5656$\times 10^{-2}$	−9.0483$\times 10^{-7}$	−	+2.1506$\times 10^{-6}$	−9.1594$\times 10^{-3}$	−
		μ-DE vs. μ-DE-ERM	≈4.0409$\times 10^{-1}$	−1.0102$\times 10^{-6}$	≈4.1512$\times 10^{-1}$	−4.4475$\times 10^{-4}$	+9.6067$\times 10^{-5}$	+3.8126$\times 10^{-3}$	≈1.6819$\times 10^{-1}$	−	+1.0168$\times 10^{-2}$	≈9.6892$\times 10^{-1}$	−
	1$\times 10^{5}$	DE vs. μ-DE	+2.4464$\times 10^{-8}$	−4.2431$\times 10^{-9}$	+8.9206$\times 10^{-9}$	−4.2431$\times 10^{-9}$	−5.4005$\times 10^{-9}$	≈6.8875$\times 10^{-2}$	+1.0898$\times 10^{-9}$	+2.1550$\times 10^{-4}$	≈3.3706$\times 10^{-1}$	+1.3745$\times 10^{-7}$	−
		DE vs. μ-DE-ERM	+6.4192$\times 10^{-10}$	−4.2431$\times 10^{-9}$	+3.9260$\times 10^{-9}$	−4.2431$\times 10^{-9}$	≈1.3015$\times 10^{-1}$	+8.5577$\times 10^{-4}$	+1.0898$\times 10^{-9}$	+1.1759$\times 10^{-6}$	+3.8547$\times 10^{-9}$	+1.3745$\times 10^{-7}$	−
		μ-DE vs. μ-DE-ERM	+1.8502$\times 10^{-4}$	−2.2949$\times 10^{-8}$	≈1.9324$\times 10^{-1}$	−5.8547$\times 10^{-9}$	+4.1072$\times 10^{-8}$	≈6.8875$\times 10^{-2}$	≈2.0014$\times 10^{-1}$	+1.5591$\times 10^{-2}$	+4.2364$\times 10^{-9}$	≈7.7908$\times 10^{-2}$	−
Shifted	1$\times 10^{3}$	DE vs. μ-DE	−1.6927$\times 10^{-9}$	−1.5127$\times 10^{-5}$	−4.2431$\times 10^{-9}$	+3.5018$\times 10^{-8}$	−2.3488$\times 10^{-2}$	−4.2431$\times 10^{-9}$	−4.2470$\times 10^{-9}$	−4.2470$\times 10^{-9}$	−4.2470$\times 10^{-9}$	−4.2470$\times 10^{-9}$	−
		DE vs. μ-DE-ERM	−1.6927$\times 10^{-9}$	−4.7900$\times 10^{-9}$	−4.5675$\times 10^{-9}$	+2.3547$\times 10^{-7}$	−2.3488$\times 10^{-2}$	−5.7910$\times 10^{-9}$	−4.2470$\times 10^{-9}$	−4.2470$\times 10^{-9}$	−4.2470$\times 10^{-9}$	−4.2470$\times 10^{-9}$	−
		μ-DE vs. μ-DE-ERM	−1.6927$\times 10^{-9}$	−2.3346$\times 10^{-8}$	≈2.0724$\times 10^{-1}$	≈8.6137$\times 10^{-1}$	≈9.9226$\times 10^{-1}$	≈4.2631$\times 10^{-1}$	−2.4712$\times 10^{-3}$	≈5.2345$\times 10^{-2}$	−2.5659$\times 10^{-2}$	≈5.0032$\times 10^{-2}$	−
	1$\times 10^{4}$	DE vs. μ-DE	−	−3.1309$\times 10^{-9}$	−	+4.1184$\times 10^{-9}$	−3.0028$\times 10^{-4}$	−1.0532$\times 10^{-7}$	−	≈8.4585$\times 10^{-1}$	+2.6957$\times 10^{-6}$	−	−
		DE vs. μ-DE-ERM	−	−1.3323$\times 10^{-9}$	−	+4.9754$\times 10^{-9}$	≈6.7655$\times 10^{-1}$	−7.6589$\times 10^{-6}$	−	≈5.0134$\times 10^{-2}$	+8.7771$\times 10^{-9}$	−	−
		μ-DE vs. μ-DE-ERM	−	−8.5217$\times 10^{-9}$	−	≈5.0021$\times 10^{-2}$	+3.5846$\times 10^{-2}$	≈4.1360$\times 10^{-1}$	−	≈5.0134$\times 10^{-2}$	+3.3458$\times 10^{-4}$	−	−
	1$\times 10^{5}$	DE vs. μ-DE	−	−	−	−4.1431$\times 10^{-10}$	−1.0740$\times 10^{-7}$	≈4.9110$\times 10^{-1}$	−	−	−	≈1.6143$\times 10^{-1}$	−
		DE vs. μ-DE-ERM	−	−	−	−2.7843$\times 10^{-10}$	≈6.2074$\times 10^{-1}$	+6.8192$\times 10^{-3}$	−	+7.1951$\times 10^{-4}$	+1.3122$\times 10^{-7}$	+1.6197$\times 10^{-3}$	−
		μ-DE vs. μ-DE-ERM	−	−	−	≈3.3706$\times 10^{-1}$	+1.0740$\times 10^{-7}$	≈1.4866$\times 10^{-1}$	−	+7.1951$\times 10^{-4}$	+1.3122$\times 10^{-7}$	+1.0631$\times 10^{-2}$	−
Rotated	1$\times 10^{3}$	DE vs. μ-DE	−4.2470$\times 10^{-9}$	+3.9022$\times 10^{-2}$	−5.4005$\times 10^{-9}$	+3.9022$\times 10^{-2}$	+1.2291$\times 10^{-6}$	−4.2470$\times 10^{-9}$	−4.2126$\times 10^{-9}$	−4.2470$\times 10^{-9}$	−4.7900$\times 10^{-9}$	−4.2470$\times 10^{-9}$	−
		DE vs. μ-DE-ERM	−4.2470$\times 10^{-9}$	≈9.0732$\times 10^{-1}$	−5.4005$\times 10^{-9}$	≈2.7034$\times 10^{-1}$	+2.0156$\times 10^{-6}$	−4.2470$\times 10^{-9}$	−4.2126$\times 10^{-9}$	−4.5934$\times 10^{-8}$	−4.7900$\times 10^{-9}$	−4.2470$\times 10^{-9}$	−
		μ-DE vs. μ-DE-ERM	≈4.6093$\times 10^{-1}$	−3.9022$\times 10^{-2}$	−3.8399$\times 10^{-3}$	≈4.3768$\times 10^{-1}$	≈9.3264$\times 10^{-2}$	−2.8077$\times 10^{-3}$	−1.2302$\times 10^{-6}$	≈4.1512$\times 10^{-1}$	≈8.1589$\times 10^{-1}$	+4.3415$\times 10^{-3}$	−
	1$\times 10^{4}$	DE vs. μ-DE	+2.3542$\times 10^{-7}$	≈8.3099$\times 10^{-1}$	−8.7672$\times 10^{-6}$	≈9.7678$\times 10^{-1}$	+3.0225$\times 10^{-5}$	−4.2317$\times 10^{-9}$	−1.6547$\times 10^{-6}$	−4.9685$\times 10^{-3}$	−3.9371$\times 10^{-2}$	−1.5039$\times 10^{-4}$	−
		DE vs. μ-DE-ERM	+5.1033$\times 10^{-7}$	−5.3957$\times 10^{-9}$	−8.7672$\times 10^{-6}$	−1.6588$\times 10^{-3}$	+3.0064$\times 10^{-7}$	−4.2317$\times 10^{-9}$	−1.5057$\times 10^{-6}$	−1.5469$\times 10^{-2}$	≈5.7976$\times 10^{-2}$	+1.6069$\times 10^{-2}$	−
		μ-DE vs. μ-DE-ERM	≈3.5666$\times 10^{-1}$	−5.3957$\times 10^{-9}$	≈9.2272$\times 10^{-1}$	−1.6588$\times 10^{-3}$	+1.5104$\times 10^{-3}$	+4.1610$\times 10^{-2}$	≈8.6898$\times 10^{-1}$	≈8.0754$\times 10^{-1}$	+3.9458$\times 10^{-5}$	+3.3840$\times 10^{-5}$	−
	1$\times 10^{5}$	DE vs. μ-DE	+4.9022$\times 10^{-5}$	−1.6547$\times 10^{-8}$	+6.1518$\times 10^{-3}$	−1.4539$\times 10^{-7}$	−2.5338$\times 10^{-5}$	−4.2393$\times 10^{-9}$	+8.7566$\times 10^{-9}$	+2.1506$\times 10^{-4}$	−2.6832$\times 10^{-8}$	+1.3398$\times 10^{-3}$	−
		DE vs. μ-DE-ERM	+3.2052$\times 10^{-7}$	−4.2431$\times 10^{-9}$	+2.8683$\times 10^{-4}$	−6.0865$\times 10^{-9}$	≈9.3248$\times 10^{-2}$	−4.2393$\times 10^{-9}$	+1.0972$\times 10^{-9}$	+1.1688$\times 10^{-6}$	+2.0041$\times 10^{-2}$	+4.2877$\times 10^{-8}$	−
		μ-DE vs. μ-DE-ERM	+1.0482$\times 10^{-5}$	−3.9965$\times 10^{-8}$	≈8.4663$\times 10^{-2}$	−7.1546$\times 10^{-8}$	+3.0325$\times 10^{-6}$	≈3.5671$\times 10^{-1}$	≈7.0494$\times 10^{-1}$	≈6.6050$\times 10^{-2}$	+1.0935$\times 10^{-9}$	+1.3584$\times 10^{-7}$	−

**Table 10:** Summary of the victories obtained by each algorithm in the Mann-Whitney U tests for the CEC 2005 benchmark problems.

Problem Type	FE	Algorithm	$f_{1}$	$f_{2}$	$f_{3}$	$f_{4}$	$f_{5}$	$f_{6}$	$f_{7}$	$f_{8}$	$f_{9}$	$f_{10}$	$f_{11}$	Total
Base	1$\times 10^{3}$	DE	0	0	0	0	0	0	0	0	0	0	0	0
		μ-DE	1	1	1	1	1	2	1	1	1	1	0	11
		μ-DE-ERM	1	1	1	1	1	1	2	1	2	2	0	13
	1$\times 10^{4}$	DE	0	0	0	0	0	0	0	0	2	0	0	2
		μ-DE	1	1	1	1	2	2	1	0	1	1	0	11
		μ-DE-ERM	1	2	1	2	0	1	1	0	0	1	0	9
	1$\times 10^{5}$	DE	2	0	2	0	0	1	2	2	1	2	0	12
		μ-DE	1	1	0	1	2	0	0	1	1	0	0	7
		μ-DE-ERM	0	2	0	2	0	0	0	0	0	0	0	4
Shifted	1$\times 10^{3}$	DE	0	0	0	2	0	0	0	0	0	0	0	2
		μ-DE	1	1	1	0	1	1	1	1	1	1	0	9
		μ-DE-ERM	2	2	1	0	1	1	2	1	2	1	0	13
	1$\times 10^{4}$	DE	0	0	0	2	0	0	0	0	2	0	0	4
		μ-DE	0	1	0	0	2	1	0	0	1	0	0	5
		μ-DE-ERM	0	2	0	0	0	1	0	0	0	0	0	3
	1$\times 10^{5}$	DE	0	0	0	0	0	1	0	1	1	1	0	4
		μ-DE	0	0	0	1	2	0	0	1	1	1	0	6
		μ-DE-ERM	0	0	0	1	0	0	0	0	0	0	0	1
Rotated	1$\times 10^{3}$	DE	0	1	0	1	2	0	0	0	0	0	0	4
		μ-DE	1	0	1	0	0	1	1	1	1	2	0	8
		μ-DE-ERM	1	1	2	0	0	2	2	1	1	1	0	11
	1$\times 10^{4}$	DE	2	0	0	0	2	0	0	0	0	1	0	5
		μ-DE	0	0	1	0	1	2	1	1	2	2	0	10
		μ-DE-ERM	0	2	1	2	0	1	1	1	0	0	0	8
	1$\times 10^{5}$	DE	2	0	2	0	0	0	2	2	1	2	0	11
		μ-DE	1	1	0	1	2	1	0	0	2	1	0	9
		μ-DE-ERM	0	2	0	2	0	1	0	0	0	0	0	5

**Table 11:** Selected benchmark problems from CEC 2017.

Problem	Name	Features
$f_{12}$	Shifted and Rotated Zakharov Function	Unimodal, non-separable
$f_{13}$	Shifted and Rotated Levy Function	Multimodal, non-separable, local optima’s number is huge
$f_{14}$	Hybrid Function 3 (N=3)	Hybrid, multimodal, non-separable, smooth but narrow ridge, local optima’s number is huge, asymmetrical, continuous everywhere yet differentiable nowhere
$f_{15}$	Composition Function 7 (N=6, shifted, and rotated)	Composite, non-separable, asymmetrical, different properties around different local optima

**Table 12:** Results obtained with μ-DE-ERM, L-SHADE, and RuGA over the CEC 2017 benchmark problems after 25 independent runs, considering evaluation budgets of 1$\times 10^{3}$, 1$\times 10^{4}$, and 1$\times 10^{5}$.

FE	Algorithm	Index	$f_{12}$	$f_{13}$	$f_{14}$	$f_{15}$
1$\times 10^{3}$	L-SHADE	Min	5.8760$\times 10^{3}$	1.1515$\times 10^{3}$	1.0519$\times 10^{7}$	4.9685$\times 10^{60}$
		Max	2.8271$\times 10^{4}$	2.3140$\times 10^{3}$	1.9541$\times 10^{8}$	1.6717$\times 10^{61}$
		Mean	1.9972$\times 10^{4}$	1.6491$\times 10^{3}$	7.5075$\times 10^{7}$	1.0707$\times 10^{61}$
		Std.	4.5261$\times 10^{3}$	3.0155$\times 10^{2}$	4.7541$\times 10^{7}$	2.8544$\times 10^{60}$
	RuGA	Min	3.4238$\times 10^{3}$	3.2727$\times 10^{1}$	2.3339$\times 10^{3}$	7.2787$\times 10^{58}$
		Max	3.2696$\times 10^{4}$	1.3512$\times 10^{3}$	8.4679$\times 10^{8}$	1.0705$\times 10^{61}$
		Mean	1.9077$\times 10^{4}$	2.8991$\times 10^{2}$	8.6945$\times 10^{7}$	1.8222$\times 10^{60}$
		Std.	8.0953$\times 10^{3}$	3.1837$\times 10^{2}$	1.9212$\times 10^{8}$	2.2626$\times 10^{60}$
	μ-DE-ERM	Min	1.6132$\times 10^{4}$	6.2863$\times 10^{2}$	1.3348$\times 10^{6}$	5.0500$\times 10^{2}$
		Max	2.2455$\times 10^{5}$	2.9535$\times 10^{3}$	1.8232$\times 10^{7}$	5.0500$\times 10^{2}$
		Mean	7.4019$\times 10^{4}$	1.3417$\times 10^{3}$	5.7545$\times 10^{6}$	5.0500$\times 10^{2}$
		Std.	4.7269$\times 10^{4}$	5.5643$\times 10^{2}$	3.7179$\times 10^{6}$	0.0000
1$\times 10^{4}$	L-SHADE	Min	5.4873$\times 10^{3}$	1.5155$\times 10^{1}$	2.8521$\times 10^{3}$	1.8033$\times 10^{58}$
		Max	1.3633$\times 10^{4}$	5.0988$\times 10^{1}$	1.0829$\times 10^{4}$	6.9673$\times 10^{58}$
		Mean	9.7353$\times 10^{3}$	2.7615$\times 10^{1}$	7.4654$\times 10^{3}$	3.3819$\times 10^{58}$
		Std.	2.1749$\times 10^{3}$	9.5247	2.2990$\times 10^{3}$	1.1467$\times 10^{58}$
	RuGA	Min	3.6308$\times 10^{3}$	9.9818$\times 10^{-1}$	1.0054$\times 10^{3}$	1.2462$\times 10^{51}$
		Max	1.9746$\times 10^{4}$	1.0640$\times 10^{2}$	5.4331$\times 10^{4}$	4.7056$\times 10^{54}$
		Mean	9.6417$\times 10^{3}$	4.2910$\times 10^{1}$	6.5033$\times 10^{3}$	8.0407$\times 10^{53}$
		Std.	4.4466$\times 10^{3}$	3.1114$\times 10^{1}$	1.0685$\times 10^{4}$	1.2331$\times 10^{54}$
	μ-DE-ERM	Min	2.3154$\times 10^{3}$	1.6680$\times 10^{-2}$	1.7803$\times 10^{1}$	5.0451$\times 10^{2}$
		Max	7.4421$\times 10^{4}$	9.1123$\times 10^{-1}$	5.8896$\times 10^{3}$	5.0500$\times 10^{2}$
		Mean	2.9994$\times 10^{4}$	2.3114$\times 10^{-1}$	1.3097$\times 10^{3}$	5.0498$\times 10^{2}$
		Std.	1.8027$\times 10^{4}$	1.9236$\times 10^{-1}$	1.5258$\times 10^{3}$	9.6047$\times 10^{-2}$
1$\times 10^{5}$	L-SHADE	Min	1.1837$\times 10^{-2}$	0.0000	0.0000	5.3711$\times 10^{35}$
		Max	4.9933$\times 10^{-1}$	0.0000	6.1370$\times 10^{-1}$	4.6930$\times 10^{37}$
		Mean	1.2821$\times 10^{-1}$	0.0000	2.4552$\times 10^{-2}$	1.3483$\times 10^{37}$
		Std.	1.3719$\times 10^{-1}$	0.0000	1.2026$\times 10^{-1}$	1.0811$\times 10^{37}$
	RuGA	Min	7.6841$\times 10^{-6}$	5.1956$\times 10^{-11}$	1.7891	6.4713$\times 10^{43}$
		Max	3.6072$\times 10^{-4}$	2.4362$\times 10^{1}$	1.2178$\times 10^{2}$	2.5508$\times 10^{49}$
		Mean	6.4784$\times 10^{-5}$	1.5050	4.8050$\times 10^{1}$	1.9425$\times 10^{48}$
		Std.	8.5888$\times 10^{-5}$	4.8258	3.6686$\times 10^{1}$	4.9943$\times 10^{48}$
	μ-DE-ERM	Min	6.5438$\times 10^{-18}$	1.4998$\times 10^{-32}$	9.2318$\times 10^{-33}$	5.0159$\times 10^{2}$
		Max	3.0500$\times 10^{-11}$	1.4998$\times 10^{-32}$	1.6322$\times 10^{1}$	5.0451$\times 10^{2}$
		Mean	2.6801$\times 10^{-12}$	1.4998$\times 10^{-32}$	6.5289$\times 10^{-1}$	5.0343$\times 10^{2}$
		Std.	7.7315$\times 10^{-12}$	5.4738$\times 10^{-48}$	3.1985	8.2406$\times 10^{-1}$

**Table 13:** Best performance counts by algorithm based on FE for the CEC 2017 benchmark problems.

FE	Algorithm	Min	Max	Mean	Std.	Total
1$\times 10^{3}$	L-SHADE	0	1	0	2	3
	RuGA	3	1	2	0	6
	μ-DE-ERM	1	2	2	2	7
1$\times 10^{4}$	L-SHADE	0	1	0	1	2
	RuGA	0	0	1	0	1
	μ-DE-ERM	4	3	3	3	13
1$\times 10^{5}$	L-SHADE	2	2	2	2	8
	RuGA	0	0	0	0	0
	μ-DE-ERM	2	2	2	2	8

**Table 14:** Results of the Kruskal-Wallis tests for the CEC 2017 benchmark problems.

FE	Index	$f_{12}$	$f_{13}$	$f_{14}$	$f_{15}$
1$\times 10^{3}$	*p*-value	2.7761$\times 10^{-9}$	3.2365$\times 10^{-11}$	9.2282$\times 10^{-7}$	2.6414$\times 10^{-15}$
	*H*	3.9404$\times 10^{1}$	4.8308$\times 10^{1}$	2.7792$\times 10^{1}$	6.7135$\times 10^{1}$
1$\times 10^{4}$	*p*-value	4.1063$\times 10^{-8}$	1.3148$\times 10^{-11}$	3.4716$\times 10^{-10}$	1.9521$\times 10^{-15}$
	*H*	3.4016$\times 10^{1}$	5.0109$\times 10^{1}$	4.3562$\times 10^{1}$	6.7740$\times 10^{1}$
1$\times 10^{5}$	*p*-value	5.1760$\times 10^{-15}$	3.7400$\times 10^{-16}$	2.7177$\times 10^{-14}$	5.1712$\times 10^{-15}$
	*H*	6.5789$\times 10^{1}$	7.1045$\times 10^{1}$	6.2473$\times 10^{1}$	6.5791$\times 10^{1}$

**Table 15:** Results of the Mann-Whitney U tests for the CEC 2017 benchmark problems.

FE	Test	$f_{12}$	$f_{13}$	$f_{14}$	$f_{15}$
1$\times 10^{3}$	L-SHADE vs. RuGA	≈6.5541$\times 10^{-1}$	−6.8572$\times 10^{-9}$	−4.3439$\times 10^{-3}$	−5.2120$\times 10^{-9}$
	L-SHADE vs. μ-DE-ERM	+1.4925$\times 10^{-7}$	−7.8561$\times 10^{-3}$	−5.4005$\times 10^{-9}$	−2.9185$\times 10^{-10}$
	RuGA vs. μ-DE-ERM	+1.7094$\times 10^{-7}$	+6.4115$\times 10^{-8}$	≈1.1160$\times 10^{-1}$	−2.9185$\times 10^{-10}$
1$\times 10^{4}$	L-SHADE vs. RuGA	≈5.2198$\times 10^{-1}$	≈1.9360$\times 10^{-1}$	−1.8057$\times 10^{-4}$	−1.4157$\times 10^{-9}$
	L-SHADE vs. μ-DE-ERM	+8.1655$\times 10^{-7}$	−4.2470$\times 10^{-9}$	−1.7564$\times 10^{-8}$	−5.6262$\times 10^{-10}$
	RuGA vs. μ-DE-ERM	+2.0217$\times 10^{-6}$	−4.2470$\times 10^{-9}$	−3.6053$\times 10^{-5}$	−5.6262$\times 10^{-10}$
1$\times 10^{5}$	L-SHADE vs. RuGA	−4.2470$\times 10^{-9}$	+1.9457$\times 10^{-10}$	+5.6262$\times 10^{-10}$	+4.2393$\times 10^{-9}$
	L-SHADE vs. μ-DE-ERM	−4.2470$\times 10^{-9}$	+8.3174$\times 10^{-12}$	+7.0056$\times 10^{-8}$	−4.2393$\times 10^{-9}$
	RuGA vs. μ-DE-ERM	−4.2470$\times 10^{-9}$	−1.9457$\times 10^{-10}$	−4.5715$\times 10^{-9}$	−4.2393$\times 10^{-9}$

**Table 16:** Summary of the victories obtained by each algorithm in the Mann-Whitney U tests for the CEC 2017 benchmark problems.

FE	Algorithm	$f_{12}$	$f_{13}$	$f_{14}$	$f_{15}$	Total
1$\times 10^{3}$	L-SHADE	1	0	0	0	1
	RuGA	1	2	1	1	5
	μ-DE-ERM	0	1	1	2	4
1$\times 10^{4}$	L-SHADE	1	0	0	0	1
	RuGA	1	0	1	1	3
	μ-DE-ERM	0	2	2	2	6
1$\times 10^{5}$	L-SHADE	0	2	2	1	5
	RuGA	1	0	0	0	1
	μ-DE-ERM	2	1	1	2	6

**Table 17:** Bounds of the box constraints in the identification and control tuning problems.

Bound	m (kg)	$L_{\mathrm{cm}}$ (m)	$I_{z}$ (kg.m^2^)
$p_{l}$	0.10125	0.075	0.00078
$p_{u}$	0.30375	0.225	0.00234
Bound	$k_{p}$	$k_{i}$	$k_{d}$
$g_{l}$	0	0	0
$g_{u}$	100	1	100

**Table 18:** Descriptive results for the robotic manipulator identification.

Algorithm	μ-DE-Cauchy				μ-DE-Shrink				μ-DE-ERM			
**Index**	m	$L_{\mathrm{cm}}$	$I_{z}$	$J_{\mathrm{si}}$	m	$L_{\mathrm{cm}}$	$I_{z}$	$J_{\mathrm{si}}$	m	$L_{\mathrm{cm}}$	$I_{z}$	$J_{\mathrm{si}}$
1	1.8043$\times 10^{-1}$	1.6234$\times 10^{-1}$	1.3567$\times 10^{-3}$	3.8709	1.7757$\times 10^{-1}$	1.7265$\times 10^{-1}$	8.1904$\times 10^{-4}$	6.9158$\times 10^{-2}$	1.8404$\times 10^{-1}$	1.5842$\times 10^{-1}$	1.5134$\times 10^{-3}$	1.3087$\times 10^{-1}$
2	1.2547$\times 10^{-1}$	2.1136$\times 10^{-1}$	8.5767$\times 10^{-4}$	1.8669$\times 10^{1}$	1.6210$\times 10^{-1}$	1.6898$\times 10^{-1}$	1.5217$\times 10^{-3}$	6.5844	2.0382$\times 10^{-1}$	1.4903$\times 10^{-1}$	1.5893$\times 10^{-3}$	1.5199$\times 10^{-7}$
3	2.0820$\times 10^{-1}$	1.5636$\times 10^{-1}$	9.9436$\times 10^{-4}$	2.4819$\times 10^{-1}$	1.8899$\times 10^{-1}$	1.4452$\times 10^{-1}$	2.2025$\times 10^{-3}$	8.6420	2.1008$\times 10^{-1}$	1.3876$\times 10^{-1}$	2.0873$\times 10^{-3}$	1.3142$\times 10^{-1}$
4	1.7597$\times 10^{-1}$	1.5878$\times 10^{-1}$	1.7106$\times 10^{-3}$	2.4979	1.2850$\times 10^{-1}$	2.1866$\times 10^{-1}$	0.0000	1.1923	2.0808$\times 10^{-1}$	1.4598$\times 10^{-1}$	1.6821$\times 10^{-3}$	1.4450$\times 10^{-9}$
5	2.9519$\times 10^{-1}$	1.2047$\times 10^{-1}$	1.7521$\times 10^{-3}$	8.4095$\times 10^{-1}$	2.4002$\times 10^{-1}$	1.2833$\times 10^{-1}$	2.1570$\times 10^{-3}$	1.9515$\times 10^{-1}$	1.8390$\times 10^{-1}$	1.6435$\times 10^{-1}$	1.1509$\times 10^{-3}$	1.4156$\times 10^{-3}$
6	1.7667$\times 10^{-1}$	1.6254$\times 10^{-1}$	1.4695$\times 10^{-3}$	3.2287$\times 10^{-1}$	1.6883$\times 10^{-1}$	1.6475$\times 10^{-1}$	1.5608$\times 10^{-3}$	3.1584	1.8309$\times 10^{-1}$	1.5834$\times 10^{-1}$	1.5439$\times 10^{-3}$	1.8096$\times 10^{-1}$
7	1.9643$\times 10^{-1}$	1.4663$\times 10^{-1}$	1.9144$\times 10^{-3}$	1.1195	3.0358$\times 10^{-1}$	1.1404$\times 10^{-1}$	2.1039$\times 10^{-3}$	5.7964$\times 10^{-1}$	1.8046$\times 10^{-1}$	1.5170$\times 10^{-1}$	2.2921$\times 10^{-3}$	1.3525$\times 10^{1}$
8	2.4266$\times 10^{-1}$	1.2868$\times 10^{-1}$	2.0857$\times 10^{-3}$	9.9135$\times 10^{-2}$	1.5881$\times 10^{-1}$	1.7441$\times 10^{-1}$	1.3163$\times 10^{-3}$	3.3597	1.9699$\times 10^{-1}$	1.4989$\times 10^{-1}$	1.7017$\times 10^{-3}$	5.4594$\times 10^{-2}$
9	2.0491$\times 10^{-1}$	1.3845$\times 10^{-1}$	2.2121$\times 10^{-3}$	6.2728$\times 10^{-1}$	2.4063$\times 10^{-1}$	1.3127$\times 10^{-1}$	1.9526$\times 10^{-3}$	2.8638$\times 10^{-1}$	1.6702$\times 10^{-1}$	1.7367$\times 10^{-1}$	1.0965$\times 10^{-3}$	1.7561$\times 10^{-1}$
10	2.6263$\times 10^{-1}$	1.2392$\times 10^{-1}$	2.0517$\times 10^{-3}$	4.3392$\times 10^{-1}$	2.5085$\times 10^{-1}$	1.2597$\times 10^{-1}$	2.1183$\times 10^{-3}$	1.8419$\times 10^{-1}$	1.6993$\times 10^{-1}$	1.7646$\times 10^{-1}$	8.3021$\times 10^{-4}$	1.0714$\times 10^{-2}$
11	1.8991$\times 10^{-1}$	1.5210$\times 10^{-1}$	1.7415$\times 10^{-3}$	2.7039$\times 10^{-1}$	1.6256$\times 10^{-1}$	1.8027$\times 10^{-1}$	8.4794$\times 10^{-4}$	1.2277$\times 10^{-1}$	1.7256$\times 10^{-1}$	1.7180$\times 10^{-1}$	1.0331$\times 10^{-3}$	3.8837$\times 10^{-2}$
12	2.1571$\times 10^{-1}$	1.4527$\times 10^{-1}$	1.5508$\times 10^{-3}$	3.1335$\times 10^{-1}$	1.3854$\times 10^{-1}$	1.9654$\times 10^{-1}$	8.0217$\times 10^{-4}$	7.7314	1.5476$\times 10^{-1}$	1.8604$\times 10^{-1}$	7.8019$\times 10^{-4}$	2.6322$\times 10^{-1}$
13	2.1955$\times 10^{-1}$	1.3705$\times 10^{-1}$	1.9973$\times 10^{-3}$	4.0955$\times 10^{-1}$	1.9723$\times 10^{-1}$	1.4680$\times 10^{-1}$	1.8820$\times 10^{-3}$	1.3973	3.0279$\times 10^{-1}$	1.1531$\times 10^{-1}$	2.0209$\times 10^{-3}$	6.3684$\times 10^{-1}$
14	2.9121$\times 10^{-1}$	1.1866$\times 10^{-1}$	1.9533$\times 10^{-3}$	1.8369	2.0264$\times 10^{-1}$	1.4310$\times 10^{-1}$	1.9827$\times 10^{-3}$	9.2968$\times 10^{-1}$	1.8360$\times 10^{-1}$	1.6647$\times 10^{-1}$	1.0256$\times 10^{-3}$	2.8682$\times 10^{-3}$
15	1.5005$\times 10^{-1}$	1.8808$\times 10^{-1}$	8.3369$\times 10^{-4}$	8.0219$\times 10^{-1}$	1.4328$\times 10^{-1}$	1.9247$\times 10^{-1}$	8.4218$\times 10^{-4}$	4.3820	1.7894$\times 10^{-1}$	1.6938$\times 10^{-1}$	9.8408$\times 10^{-4}$	6.2348$\times 10^{-2}$
16	1.8753$\times 10^{-1}$	1.5783$\times 10^{-1}$	1.4557$\times 10^{-3}$	3.3789$\times 10^{-1}$	1.4364$\times 10^{-1}$	1.8597$\times 10^{-1}$	1.1991$\times 10^{-3}$	9.5185	1.6404$\times 10^{-1}$	1.8053$\times 10^{-1}$	7.8056$\times 10^{-4}$	4.2827$\times 10^{-2}$
17	1.7706$\times 10^{-1}$	1.6389$\times 10^{-1}$	1.6591$\times 10^{-3}$	3.0116$\times 10^{1}$	1.9744$\times 10^{-1}$	1.6103$\times 10^{-1}$	9.7541$\times 10^{-4}$	2.5385$\times 10^{-1}$	1.5818$\times 10^{-1}$	1.8085$\times 10^{-1}$	9.6464$\times 10^{-4}$	3.6878$\times 10^{-1}$
18	1.4008$\times 10^{-1}$	1.9389$\times 10^{-1}$	8.9031$\times 10^{-4}$	7.9287	1.8883$\times 10^{-1}$	1.6712$\times 10^{-1}$	8.2347$\times 10^{-4}$	5.4316	2.3832$\times 10^{-1}$	1.2755$\times 10^{-1}$	2.2389$\times 10^{-3}$	1.8832$\times 10^{-4}$
19	3.0071$\times 10^{-1}$	1.2980$\times 10^{-1}$	9.1176$\times 10^{-4}$	1.8958	1.7696$\times 10^{-1}$	1.6594$\times 10^{-1}$	1.2561$\times 10^{-3}$	2.0854$\times 10^{-1}$	2.3281$\times 10^{-1}$	1.3047$\times 10^{-1}$	2.1532$\times 10^{-3}$	6.5166$\times 10^{-10}$
20	1.5512$\times 10^{-1}$	1.8292$\times 10^{-1}$	9.5024$\times 10^{-4}$	5.8783$\times 10^{-1}$	1.9470$\times 10^{-1}$	1.4489$\times 10^{-1}$	2.0558$\times 10^{-3}$	9.6063$\times 10^{-1}$	2.1487$\times 10^{-1}$	1.4125$\times 10^{-1}$	1.8297$\times 10^{-3}$	5.8670$\times 10^{-5}$
21	2.0401$\times 10^{-1}$	1.3878$\times 10^{-1}$	2.2111$\times 10^{-3}$	6.5593$\times 10^{-1}$	1.3981$\times 10^{-1}$	1.9607$\times 10^{-1}$	7.8116$\times 10^{-4}$	7.0000	1.5264$\times 10^{-1}$	1.8636$\times 10^{-1}$	8.3880$\times 10^{-4}$	4.9955$\times 10^{-1}$
22	1.7099$\times 10^{-1}$	1.7387$\times 10^{-1}$	9.5500$\times 10^{-4}$	2.3734$\times 10^{-1}$	2.2815$\times 10^{-1}$	1.3234$\times 10^{-1}$	2.1246$\times 10^{-3}$	2.3288	1.9298$\times 10^{-1}$	1.5682$\times 10^{-1}$	1.3721$\times 10^{-3}$	2.5362$\times 10^{-3}$
23	2.8853$\times 10^{-1}$	1.1491$\times 10^{-1}$	2.2654$\times 10^{-3}$	2.8760$\times 10^{-1}$	2.0307$\times 10^{-1}$	1.3763$\times 10^{-1}$	2.3056$\times 10^{-3}$	8.3769	1.9497$\times 10^{-1}$	1.5127$\times 10^{-1}$	1.6665$\times 10^{-3}$	5.9739$\times 10^{-2}$
24	1.9193$\times 10^{-1}$	1.4557$\times 10^{-1}$	2.0784$\times 10^{-3}$	1.8103	1.4764$\times 10^{-1}$	1.8778$\times 10^{-1}$	9.4120$\times 10^{-4}$	3.1438	1.9621$\times 10^{-1}$	1.5486$\times 10^{-1}$	1.4106$\times 10^{-3}$	1.6200$\times 10^{-3}$
25	1.3853$\times 10^{-1}$	1.9648$\times 10^{-1}$	8.0783$\times 10^{-4}$	7.5118	1.3718$\times 10^{-1}$	1.9756$\times 10^{-1}$	8.0388$\times 10^{-4}$	8.3273	1.9241$\times 10^{-1}$	1.5690$\times 10^{-1}$	1.3819$\times 10^{-3}$	3.0895$\times 10^{-3}$
ine Min	1.2547$\times 10^{-1}$	1.1491$\times 10^{-1}$	8.0783$\times 10^{-4}$	9.9135$\times 10^{-2}$	1.2850$\times 10^{-1}$	1.1404$\times 10^{-1}$	0.0000	6.9158$\times 10^{-2}$	1.5264$\times 10^{-1}$	1.1531$\times 10^{-1}$	7.8019$\times 10^{-4}$	6.5166$\times 10^{-10}$
Max	3.0071$\times 10^{-1}$	2.1136$\times 10^{-1}$	2.2654$\times 10^{-3}$	3.0116$\times 10^{1}$	3.0358$\times 10^{-1}$	2.1866$\times 10^{-1}$	2.3056$\times 10^{-3}$	9.5185	3.0279$\times 10^{-1}$	1.8636$\times 10^{-1}$	2.2921$\times 10^{-3}$	1.3525$\times 10^{1}$
Mean	2.0358$\times 10^{-1}$	1.5395$\times 10^{-1}$	1.5466$\times 10^{-3}$	3.3493	1.8486$\times 10^{-1}$	1.6316$\times 10^{-1}$	1.4150$\times 10^{-3}$	3.3746	1.9270$\times 10^{-1}$	1.5770$\times 10^{-1}$	1.4387$\times 10^{-3}$	6.4771$\times 10^{-1}$
Std.	5.0892$\times 10^{-2}$	2.6110$\times 10^{-2}$	5.1139$\times 10^{-4}$	6.8635	4.2851$\times 10^{-2}$	2.7385$\times 10^{-2}$	6.3628$\times 10^{-4}$	3.3122	3.1824$\times 10^{-2}$	1.8464$\times 10^{-2}$	4.8231$\times 10^{-4}$	2.6879

**Table 19:** Mean computation time required by the algorithms to solve the identification problem.

Algorithm	Mean Time	Mean Time/Generation
μ-DE-Cauchy	18.2224 (s)	6.0741 (ms)
μ-DE-Shrink	18.0948 (s)	6.0316 (ms)
μ-DE-ERM	17.9255 (s)	5.9751 (ms)

**Table 20:** Results of the Mann-Whitney U tests for the identification problem.

Test	Adjusted *p*-Value
μ-DE-Cauchy vs. μ-DE-Shrink	≈3.6180$\times 10^{-1}$
μ-DE-Cauchy vs. μ-DE-ERM	−3.0325$\times 10^{-6}$
μ-DE-Shrink vs. μ-DE-ERM	−3.0325$\times 10^{-6}$

**Table 21:** Descriptive results for the PID controller tuning.

Algorithm	μ-DE-Cauchy				μ-DE-Shrink				μ-DE-ERM			
**Index**	$k_{p}$	$k_{i}$	$k_{d}$	$J_{\mathrm{ct}}$	$k_{p}$	$k_{i}$	$k_{d}$	$J_{\mathrm{ct}}$	$k_{p}$	$k_{i}$	$k_{d}$	$J_{\mathrm{ct}}$
1	6.7074	9.0508$\times 10^{-2}$	6.6141	5.5681	9.5990	6.8354$\times 10^{-2}$	9.2432	5.5700	9.9816	7.7708$\times 10^{-2}$	9.8745	5.5679
2	4.5651	1.0177$\times 10^{-1}$	4.4506	5.5686	1.0312$\times 10^{1}$	1.5899$\times 10^{-2}$	9.9597	5.5695	9.7484	8.8371$\times 10^{-2}$	9.6893	5.5679
3	7.9090	1.4231$\times 10^{-1}$	8.1161	5.5710	9.1896	6.2507$\times 10^{-2}$	8.9696	5.5684	8.8945	8.2458$\times 10^{-2}$	8.8022	5.5679
4	9.7263	4.9699$\times 10^{-2}$	9.5779	5.5681	8.9142	1.4740$\times 10^{-1}$	9.1002	5.5700	1.0005$\times 10^{1}$	8.0708$\times 10^{-2}$	9.9153	5.5679
5	8.9759	5.9755$\times 10^{-2}$	8.8966	5.5680	2.8966	1.2338$\times 10^{-1}$	2.9453	5.5734	9.0765	8.6429$\times 10^{-2}$	8.9999	5.5679
6	3.4038	8.8814$\times 10^{-2}$	3.3411	5.5691	9.3613	7.5450$\times 10^{-2}$	9.2193	5.5680	7.3072	9.1170$\times 10^{-2}$	7.2264	5.5680
7	8.0663	1.1958$\times 10^{-1}$	8.0731	5.5683	9.5915	4.2010$\times 10^{-2}$	9.4361	5.5682	9.7357	7.5112$\times 10^{-2}$	9.6311	5.5679
8	9.1502	1.1718$\times 10^{-1}$	9.2430	5.5688	6.7445	7.7809$\times 10^{-2}$	6.7495	5.5686	9.9259	7.9905$\times 10^{-2}$	9.8559	5.5679
9	8.7218	9.7160$\times 10^{-2}$	8.6979	5.5680	8.5038	2.7449$\times 10^{-2}$	8.2614	5.5690	8.5983	8.1142$\times 10^{-2}$	8.4942	5.5679
10	8.9542	4.0712$\times 10^{-2}$	8.7937	5.5683	9.1394	7.5477$\times 10^{-2}$	9.0077	5.5680	1.0040$\times 10^{1}$	6.7830$\times 10^{-2}$	9.9445	5.5679
11	9.9965	0.0000	9.7482	5.5689	3.6036	1.1786$\times 10^{-1}$	3.4897	5.5697	8.2894	8.2861$\times 10^{-2}$	8.2137	5.5680
12	9.6360	8.3001$\times 10^{-2}$	9.5169	5.5679	4.8910	8.7229$\times 10^{-2}$	4.8062	5.5683	9.6682	8.1347$\times 10^{-2}$	9.5803	5.5679
13	8.7568	4.1303$\times 10^{-2}$	8.4841	5.5691	8.7341	5.8181$\times 10^{-2}$	8.5213	5.5685	9.6062	8.5288$\times 10^{-2}$	9.5163	5.5679
14	7.5215	1.0872$\times 10^{-1}$	7.4939	5.5681	9.6560	0.0000	9.7554	5.5708	1.0077$\times 10^{1}$	4.8790$\times 10^{-2}$	9.9574	5.5680
15	5.7040	5.5110$\times 10^{-2}$	5.5787	5.5688	4.8364	8.4168$\times 10^{-2}$	4.7286	5.5684	8.6072	8.3721$\times 10^{-2}$	8.5127	5.5679
16	6.4277	7.3244$\times 10^{-2}$	6.2426	5.5687	8.0948	4.3071$\times 10^{-2}$	7.8472	5.5690	6.7041	9.3610$\times 10^{-2}$	6.6325	5.5681
17	9.4397	5.0405$\times 10^{-2}$	9.3096	5.5681	8.2781	1.3334$\times 10^{-1}$	8.3627	5.5689	9.3678	8.5337$\times 10^{-2}$	9.2846	5.5679
18	8.8124	5.1198$\times 10^{-2}$	8.7596	5.5682	7.4315	4.7044$\times 10^{-2}$	7.3455	5.5685	1.0035$\times 10^{1}$	7.5263$\times 10^{-2}$	9.9287	5.5679
19	8.3222	8.3605$\times 10^{-2}$	8.2221	5.5680	8.0904	0.0000	7.6373	5.5733	8.4522	9.8047$\times 10^{-2}$	8.3733	5.5680
20	8.6937	0.0000	8.5355	5.5692	8.1265	9.4746$\times 10^{-2}$	7.9165	5.5687	7.9722	1.4768$\times 10^{-1}$	8.0083	5.5688
21	7.2375	9.2622$\times 10^{-2}$	7.1665	5.5680	8.7928	1.2999$\times 10^{-1}$	8.7211	5.5683	7.3154	8.7668$\times 10^{-2}$	7.2282	5.5680
22	3.7655	0.0000	3.3768	5.5895	9.8123	2.4628$\times 10^{-1}$	9.7446	5.5717	9.3670	9.8963$\times 10^{-2}$	9.2654	5.5680
23	4.5356	8.3113$\times 10^{-2}$	4.3564	5.5697	9.2574	4.9876$\times 10^{-2}$	9.0423	5.5684	9.5175	8.3605$\times 10^{-2}$	9.4460	5.5679
24	7.1720	9.1761$\times 10^{-2}$	7.0362	5.5682	6.6589	7.2224$\times 10^{-2}$	6.5458	5.5682	8.7799	8.6591$\times 10^{-2}$	8.6950	5.5679
25	8.8732	9.2456$\times 10^{-2}$	8.7992	5.5679	9.3385	9.4519$\times 10^{-2}$	9.3427	5.5681	8.8880	8.0524$\times 10^{-2}$	8.8201	5.5679
ine Min	3.4038	0.0000	3.3411	5.5679	2.8966	0.0000	2.9453	5.5680	6.7041	4.8790$\times 10^{-2}$	6.6325	5.5679
Max	9.9965	1.4231$\times 10^{-1}$	9.7482	5.5895	1.0312$\times 10^{1}$	2.4628$\times 10^{-1}$	9.9597	5.5734	1.0077$\times 10^{1}$	1.4768$\times 10^{-1}$	9.9574	5.5688
Mean	7.6430	7.2561$\times 10^{-2}$	7.5372	5.5694	7.9942	7.8970$\times 10^{-2}$	7.8680	5.5693	9.0384	8.5205$\times 10^{-2}$	8.9558	5.5680
Std.	1.9213	3.7446$\times 10^{-2}$	1.9492	4.2481$\times 10^{-3}$	1.9949	5.2764$\times 10^{-2}$	1.9657	1.5262$\times 10^{-3}$	9.5286$\times 10^{-1}$	1.6313$\times 10^{-2}$	9.4267$\times 10^{-1}$	1.8451$\times 10^{-4}$

**Table 22:** Mean computation time required by the algorithms to solve the controller tuning problem.

Algorithm	Mean Time	Mean Time/Generation
μ-DE-Cauchy	0.2257 (s)	4.5132 (ms)
μ-DE-Shrink	0.2173 (s)	4.3470 (ms)
μ-DE-ERM	0.2210 (s)	4.4200 (ms)

**Table 23:** Results of the Mann-Whitney U tests for the controller tuning problem.

Test	Adjusted *p*-Value
μ-DE-Cauchy vs. μ-DE-Shrink	≈7.1158$\times 10^{-2}$
μ-DE-Cauchy vs. μ-DE-ERM	−5.4437$\times 10^{-7}$
μ-DE-Shrink vs. μ-DE-ERM	−5.5067$\times 10^{-8}$

**Figure A1:**
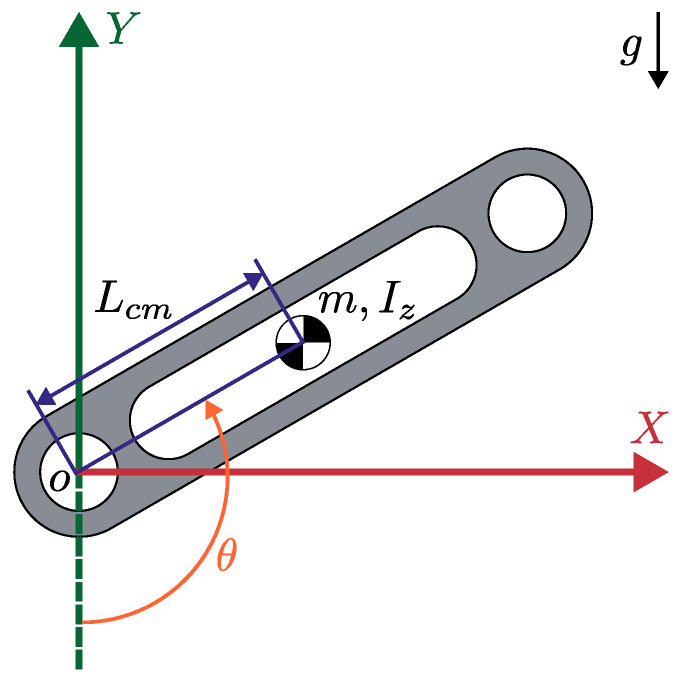
Schematic diagram of the robotic manipulator with one degree of freedom.

## Data Availability

Data will be made available on request.

## Appendix A. Benchmark Optimization Problems

This appendix details the optimization problems used as benchmarks for evaluating the μ-DE-ERM algorithm in Section 4.1. In each problem, the objective is to minimize the value of the objective function $f_{k}$, where the vector of design variables is given by $p=\left[p_{1},p_{2},\ldots ,p_{D}\right]\in R^{D}$. To add challenging characteristics to each problem, a variable transformation is performed. This transformation is defined as $z=R\;\cdot \;\left(p-s\right)\in R^{D}$, where $s\in R^{D}$ is the shift vector that fixes the location of the global optimum and $R\in R^{D\times D}$ is the rotation matrix, responsible for modifying the orientation of the search space. In addition, the term $b_{k}$ corresponds to the bias associated with the objective function $f_{k}$, vertically shifting its minimum value.

Thus, when the problems do not have the global minimum displaced s=[0,0,…,0] and $b_{k}=0$. Similarly, if the problems do not have the search space rotated around the global optimum, $R=I_{D}$, with $I_{D}$ as an identity matrix of size D×D. Then, the global minimum of all problems is found at $p^{*}=s$, and the value of the objective function at that point is $f_{k}\left(p^{*}\right)=b_{k}$.

For the problems considered, D=10 and the information on *s*, *A*, *B*, $\bar{\alpha }$ and *R*, as appropriate, is obtained from [52], while $b_{k}$ is explicitly indicated in the problem definitions for the CEC 2005 Special Session on Real-Parameter Optimization [43].

### Appendix A.1. Sphere Function (f_*1*_)

(A1) $$f_{1}\left(p\right)=\sum_{i=0}^{D}z_{i}^{2}+b_{1}$$

with $p\in {\left[-100,100\right]}^{D}$ and $b_{1}=-450$, the last for the shifted problem.

### Appendix A.2. Schwefel’s Function No. 1.2 (f_*2*_)

(A2) $$f_{2}\left(p\right)=\sum_{i=1}^{D}{\left(\sum_{j=1}^{i}z_{j}\right)}^{2}+b_{2}$$

with $p\in {\left[-100,100\right]}^{D}$ and $b_{2}=-450$, the last for the shifted problem.

### Appendix A.3. Elliptic Function (f_*3*_)

(A3) $$f_{3}\left(p\right)=\sum_{i=1}^{D}{\left(10^{6}\right)}^{\frac{i-1}{D-1}}z_{i}^{2}+b_{3}$$

with $p\in {\left[-100,100\right]}^{D}$ and $b_{3}=-450$, the last for the shifted problem.

### Appendix A.4. Schwefel’s Function No. 1.2 with Noise (f_*4*_)

(A4) $$f_{4}\left(p\right)=\left(\sum_{i=1}^{D}{\left(\sum_{j=1}^{i}z_{j}\right)}^{2}\right)\left(1+0.4|N\left(0,1\right)|\right)+b_{4}$$

with $p\in {\left[-100,100\right]}^{D}$, $N\left(0,1\right)$ denotes a normally distributed random variable with zero mean and unit variance, and $b_{4}=-450$, the last for the shifted problem.

### Appendix A.5. Schwefel’s Function No. 2.6 (f_*5*_)

(A5) $$f_{5}\left(p\right)=max\left\{|A_{i}z-B_{i}|\right\}+b_{5}$$

with $p\in {\left[-100,100\right]}^{D}$, $A\in R^{D\times D}$, $B_{i}=A_{i}\;\cdot \;s\in R^{1\times D}$ with $A_{i}\in R^{1\times D}$ as the $i^{\mathrm{th}}$ row of *A*, and $b_{5}=-310$, the last for the shifted problem.

### Appendix A.6. Rosenbrock’s Function (f_*6*_)

(A6) $$f_{6}\left(p\right)=\sum_{i=1}^{D-1}\left(100{\left({\bar{z}}_{i}^{2}-{\bar{z}}_{i+1}\right)}^{2}+{\left({\bar{z}}_{i}-1\right)}^{2}\right)+b_{6}$$

with $p\in {\left[-100,100\right]}^{D}$, $\bar{z}=R\;\cdot \;\left(R^{-1}\;\cdot \;z+1\right)$ and $b_{6}=390$, the last for the shifted problem.

### Appendix A.7. Griewank’s Function (f_*7*_)

(A7) $$f_{7}\left(p\right)=\sum_{i=1}^{D}\frac{z_{i}^{2}}{4000}-\prod_{i=1}^{D}\cos\left(\frac{z_{i}}{\sqrt{i}}\right)+1+b_{7}$$

with $p\in {\left[0,600\right]}^{D}$ and $b_{7}=-180$, the last for the shifted problem.

### Appendix A.8. Ackley’s Function (f_*8*_)

(A8) $$f_{8}\left(p\right)=-20\exp\left(-0.2\sqrt{\frac{1}{D}\sum_{i=1}^{D}z_{i}^{2}}\right)-\exp\left(\frac{1}{D}\sum_{i=1}^{D}\cos\left(2\pi z_{i}\right)\right)+20+e+b_{8}$$

with $p\in {\left[-32,32\right]}^{D}$, the Euler’s number e=2.7182 and $b_{8}=-140$, the last for the shifted problem.

### Appendix A.9. Rastrigin’s Function (f_*9*_)

(A9) $$f_{9}\left(p\right)=\sum_{i=1}^{D}\left(z_{i}^{2}-10\cos\left(2\pi z_{i}\right)+10\right)+b_{9}$$

with $p\in {\left[-5,5\right]}^{D}$ and $b_{9}=-330$, the last for the shifted problem.

### Appendix A.10. Weierstrass Function (f_*10*_)

(A10) $$f_{10}\left(p\right)=\sum_{i=1}^{D}\left(\sum_{k=0}^{k_{max}}\left[{\hat{a}}^{k}\cos\left(2\pi {\hat{b}}^{k}\left(z_{i}+0.5\right)\right)\right]\right)-D\sum_{k=0}^{k_{max}}\left[{\hat{a}}^{k}\cos\left(\pi {\hat{b}}^{k}\right)\right]+b_{10}$$

with $p\in {\left[-0.5,0.5\right]}^{D}$, $\hat{a}=0.5$, $\hat{b}=3$, $k_{max}=20$, and $b_{10}=90$, the last for the shifted problem.

### Appendix A.11. Schwefel’s Problem No. 2.13 (f_*11*_)

(A11) $$f_{11}\left(p\right)=\sum_{j=0}^{D}\left(A_{ij}\sin\left({\bar{\alpha }}_{j}\right)+B_{ij}\cos\left({\bar{\alpha }}_{j}\right)\right)-\sum_{j=0}^{D}\left(A_{ij}\sin\left(x_{j}\right)+B_{ij}\cos\left(x_{j}\right)\right)+b_{11}$$

with $p\in {\left[-\pi ,\pi \right]}^{D}$, $A\in R^{D\times D}$, $B\in R^{D\times D}$ with $A_{ij}$ and $B_{ij}$ as the corresponding component (i,j), $\bar{\alpha }\in R^{D}$, and $b_{11}=-460$, the last for the shifted problem.

## Appendix B. The Robotic Manipulator with One Degree of Freedom

The robotic manipulator with one degree of freedom, used as a case study for optimization problems related to system identification and controller tuning, is represented by the schematic diagram in Figure A1. This system consists of a link of arbitrary length *L* with one end coupled to the static origin *o* by means of an actuated revolute joint. The angular position of the link is given by θ and is measured from the −Y axis in a counterclockwise direction. For this system, the mass of the link is m=0.2025(kg), its moment of inertia is $I_{z}=0.00156\;\left(\mathrm{kg}m^{2}\right)$, and the length to the center of mass, measured from *o*, is $L_{cm}=0.15\;\left(m\right)$. The above parameters belong to an experimental laboratory prototype. It should be noted that the manipulator is affected by the acceleration due to gravity $g=9.81\;(m/s^{2})$.

### Appendix B.1. Dynamic Model and Simulation

The dynamic behavior of the robotic manipulator is described by the motion equation in (A12), with τ as the generalized torque. This equation was derived using the Euler-Lagrange methodology [53].

(A12) $$mL_{cm}^{2}\ddot{\theta }+I_{z}\ddot{\theta }+mgL_{cm}\sin\left(\theta \right)=\tau$$

The dynamics of the manipulator (A12) in state space have the form in (A13) and (A14), with $x\left(t\right)={\left[\theta \left(t\right),\dot{\theta }\left(t\right)\right]}^{T}$ as the state vector, y(t)=x(t) as the output vector (assuming that the output is given by all states in x(t) and these are measurable or observable), and $\bar{u}\left(t\right)=\tau \left(t\right)$ as the control input, with a maximum absolute value of $u_{max}=2.5\;\left(N.m\right)$.

(A13) $$\begin{array}{c} \dot{x}\left(t\right)=f\left(x\left(t\right),u\left(t\right)\right)\\ y(t)=x(t) \end{array}$$

with:

(A14) $$f\left(x\left(t\right),\bar{u}\left(t\right)\right)=\left[\begin{array}{c} \dot{\theta }\\ \frac{\bar{u}-mgL_{cm}\sin\left(\theta \right)}{mL_{cm}^{2}+I_{z}} \end{array}\right]$$

The ordinary differential equation of the model in state space in (A13) and (A14) can be solved using some numerical integration method based on an initial condition $x_{0}={\left[\theta_{0},{\dot{\theta }}_{0}\right]}^{T}$ to predict the future states of the system at discrete time intervals dt. The Euler method is adopted in this work for its simplicity and computational efficiency.

### Appendix B.2. Proportional Integral Derivative Controller

The proportional integral derivative controller in (A15) is chosen to perform the position control task with the robotic manipulator, with $k_{p}$ and $k_{d}$ as the positive proportional and derivative gains, and $e\left(t\right)=\theta_{d}\left(t\right)-\theta \left(t\right)$ as the error between the desired angular position $\theta_{d}\left(t\right)$ and the current angular position θ(t).

(A15) $$\bar{u}\left(t\right)=k_{p}e\left(t\right)+k_{p}\int e\left(t\right)\;dt+k_{d}\dot{e}\left(t\right)$$
